# Onasemnogene abeparvovec for presymptomatic infants with two copies of *SMN2* at risk for spinal muscular atrophy type 1: the Phase III SPR1NT trial

**DOI:** 10.1038/s41591-022-01866-4

**Published:** 2022-06-17

**Authors:** Kevin A. Strauss, Michelle A. Farrar, Francesco Muntoni, Kayoko Saito, Jerry R. Mendell, Laurent Servais, Hugh J. McMillan, Richard S. Finkel, Kathryn J. Swoboda, Jennifer M. Kwon, Craig M. Zaidman, Claudia A. Chiriboga, Susan T. Iannaccone, Jena M. Krueger, Julie A. Parsons, Perry B. Shieh, Sarah Kavanagh, Sitra Tauscher-Wisniewski, Bryan E. McGill, Thomas A. Macek

**Affiliations:** 1 Clinic for Special Children, Strasburg, PA USA; 2 Penn Medicine-Lancaster General Hospital, Lancaster, PA USA; 3 Departments of Pediatrics and Molecular, Cell & Cancer Biology, University of Massachusetts School of Medicine, Worcester, MA USA; 4 Department of Neurology, Sydney Children’s Hospital Network, Sydney, New South Wales Australia; 5 School of Clinical Medicine, UNSW Medicine and Health, UNSW Sydney, Sydney, New South Wales Australia; 6 The Dubowitz Neuromuscular Centre, University College London, Great Ormond Street Institute of Child Health & Great Ormond Street Hospital, London, UK; 7 National Institute of Health Research, Great Ormond Street Hospital Biomedical Research Centre, London, UK; 8 Institute of Medical Genetics, Tokyo Women’s Medical University, Tokyo, Japan; 9 Center for Gene Therapy, Nationwide Children’s Hospital, Columbus, OH USA; 10 Department of Pediatrics and Department of Neurology, The Ohio State University, Columbus, OH USA; 11 Department of Paediatrics, MDUK Oxford Neuromuscular Centre, Oxford, UK; 12 Neuromuscular Reference Center, Department of Pediatrics, CHU & University of Liège, Liège, Belgium; 13 Departments of Pediatrics, Neurology & Neurosurgery, Montreal Children’s Hospital, McGill University Health Centre, Montreal, Quebec Canada; 14 Department of Pediatrics, Nemours Children’s Hospital, Orlando, FL USA; 15 Center for Experimental Neurotherapeutics, St. Jude Children’s Research Hospital, Memphis, TN USA; 16 Department of Neurology, Massachusetts General Hospital, Boston, MA USA; 17 Department of Neurology, University of Wisconsin School of Medicine and Public Health, Madison, WI USA; 18 Washington University School of Medicine, St. Louis, MO USA; 19 Division of Pediatric Neurology, Columbia University Medical Center, New York, NY USA; 20 Department of Pediatrics, University of Texas Southwestern Medical Center, Dallas, TX USA; 21 Department of Neurology, Helen DeVos Children’s Hospital, Grand Rapids, MI USA; 22 Department of Pediatrics, University of Colorado School of Medicine, Aurora, CO USA; 23 Department of Neurology, David Geffen School of Medicine at UCLA, Los Angeles, CA USA; 24 Novartis Gene Therapies, Inc., Bannockburn, IL USA; 25 Translational Medicine, Novartis Institutes for BioMedical Research, Cambridge, MA USA

**Keywords:** Gene therapy, Development

## Abstract

SPR1NT (NCT03505099) was a Phase III, multicenter, single-arm study to investigate the efficacy and safety of onasemnogene abeparvovec for presymptomatic children with biallelic *SMN1* mutations treated at ≤6 weeks of life. Here, we report final results for 14 children with two copies of *SMN2*, expected to develop spinal muscular atrophy (SMA) type 1. Efficacy was compared with a matched Pediatric Neuromuscular Clinical Research natural-history cohort (*n* = 23). All 14 enrolled infants sat independently for ≥30 seconds at any visit ≤18 months (Bayley-III item #26; *P* < 0.001; 11 within the normal developmental window). All survived without permanent ventilation at 14 months as per protocol; 13 maintained body weight (≥3rd WHO percentile) through 18 months. No child used nutritional or respiratory support. No serious adverse events were considered related to treatment by the investigator. Onasemnogene abeparvovec was effective and well-tolerated for children expected to develop SMA type 1, highlighting the urgency for universal newborn screening.

## Abstract (web summary)

For presymptomatic infants at risk for SMA type 1, onasemnogene abeparvovec improves motor outcomes, ventilator-free survival, and nutritional/respiratory independence compared with untreated or treated symptomatic patients

## Main

Spinal muscular atrophy (SMA) results from biallelic deletions or mutations of *SMN1*, which encodes the survival motor neuron (SMN) protein essential for the development and viability of motor neurons in the ventral spinal cord^1^. *SMN2*, a gene homologous to *SMN1*, produces minimal SMN protein and exists in multiple copies in humans^2^. *SMN2* copy number correlates with the onset and severity of SMA^3^. Two copies are 79% predictive of severe, infantile-onset SMA type 1, three copies are 54% predictive of intermediate severity SMA type 2, and four copies are 88% predictive of a milder SMA type 3 phenotype with later onset^3^.

The classic clinical presentation of untreated SMA type 1 is characterized by onset of flaccid weakness and motor regression within the first 6 months of life, followed by progressive muscle wasting, dysphagia, respiratory failure, and untimely death^4–7^. Two prospective observational studies (Pediatric Neuromuscular Clinical Research [PNCR] and NeuroNEXT)^7,8^ charted the natural course of SMA type 1 and delineated meaningful trial endpoints, including survival. Untreated children do not achieve or maintain a Children’s Hospital of Philadelphia Infant Test of Neuromuscular Disorders (CHOP INTEND; range of 0–64, with lower scores indicating reduced motor function) score of ≥40 after age 6 months, and none achieve independent sitting or more advanced motor milestones. Median ventilator-free survival of patients with SMA type 1 (two *SMN2* copies) is between 8 months (NeuroNEXT) and 10.5 months (PNCR); most children who survive to 18 months require non-oral feeding support, and 100% die or require permanent ventilation by 2 years of age. Untreated patients with SMA type 2 sit independently but do not walk, whereas untreated patients with SMA type 3 develop independent walking.

There are now three approved therapies for SMA. Two augment production of intact SMN protein from each *SMN2* copy^9^ but require repeated intrathecal (nusinersen) or oral (risdiplam) dosing. Onasemnogene abeparvovec is a genetically engineered adeno-associated virus type 9 (AAV9) vector designed to express SMN protein in tissues following one-time intravenous infusion^10^. Intravenous onasemnogene abeparvovec traverses the blood–brain barrier to transfect neurons and glia throughout the nervous system, and also transfects muscle, liver, and other peripheral tissues^11,12^. Onasemnogene abeparvovec is a recombinant self-complementary AAV9 containing a human *SMN* transgene under the control of a chicken β-actin promoter and cytomegalovirus enhancer, which together ensure rapid and sustained transcription of *SMN* messenger RNA.

In two Phase III clinical trials of one-time intravenous onasemnogene abeparvovec infusion, patients with symptomatic SMA type 1 who were younger than 6 months of age were treated with 1.1 × 10^14^ vector genomes (vg)/kg (STR1VE-US, *n* = 22; STR1VE-EU, *n* = 33)^13,14^. Both studies provide evidence that *SMN* gene replacement via intravenous onasemnogene abeparvovec improves survival and motor development for patients with SMA type 1. At age 14 months, 91% (STR1VE-US) and 97% (STR1VE-EU) of treated patients were alive and free from permanent ventilation, as compared with 26% in the historical PNCR cohort. Rapid and sustained improvements in motor function were observed in both trials: (1) CHOP INTEND scores reached or exceeded 40 for 21 (95%) patients in STR1VE-US and 24 (73%) in STR1VE-EU; (2) many patients sat independently by 18 months of age (14 of 22 (64%) for ≥30 seconds (Bayley #26) in STR1VE-US and 14 of 32 (44%) for ≥10 seconds (World Health Organization; WHO) in STR1VE-EU); and (3) one patient from each study walked independently for at least five steps by 18 months of age (5%, STR1VE-US and 3%, STR1VE-EU [Bayley #43]). Similar motor milestone gains have been replicated in patients treated with intravenous onasemnogene abeparvovec in real-world settings^15^ (unpublished data, Servais, L., Day, J.W., De Vivo, D.C., Mercuri, E. & Muntoni, F).

A recent analysis summarized onasemnogene abeparvovec safety data from seven clinical trials (*n* = 102) as well as post-marketing reports (*n* = 665) through 12 November 2020^16^. In clinical trials, liver transaminases increased transiently in 90 of 102 (90%) patients and, in some cases, exceeded three times the upper limit of normal (ULN) (9% mild ≥3× ULN to <5× ULN; 6% ≥5× to <20× ULN; and 5% ≥20× ULN)^17^. Hepatotoxicity events resolved over time with prednisolone treatment. Transient decreases in platelets (<75,000 cells/µL) were also observed after vector administration^16^. In the post-marketing setting, transient hepatotoxicity, including four cases of acute liver failure, was the most common adverse event (AE). In addition, thrombotic microangiopathy (TMA) was observed in the post-marketing setting^16^. Although not observed clinically, cardiac thrombi and dorsal root ganglia toxicities were observed in nonclinical toxicology studies^16^. From these data, the study sponsor (Novartis Gene Therapies) identified five categories of potential AEs of special interest (AESIs), which include hepatotoxicity, thrombocytopenia, cardiac events, TMA, and sensory abnormalities suggestive of ganglionopathy. Overall, onasemnogene abeparvovec has demonstrated a favorable benefit–risk profile for patients with SMA who are younger than 2 years of age^13,14,18–20^. However, data covering its administration during the presymptomatic neonatal period have not been systematically collected or reported until now.

The objective of SPR1NT was to evaluate the efficacy and safety of onasemnogene abeparvovec for children with genetically confirmed SMA prior to clinical disease onset, based on the hypothesis that earlier administration of *SMN* gene therapy results in better outcomes^21^. Data from Phase I START^18,19^ and Phase III STR1VE^13,14^ studies provide some support for this hypothesis. Infants in START with baseline CHOP INTEND scores greater than 20 who received gene therapy before 3 months of age were the earliest to sit independently, and two patients who achieved independent walking were treated prior to age 3 months and had a baseline CHOP INTEND score >40 ^18,22^. Similarly, greater efficacy of other SMA disease-modifying treatments has been observed when administered earlier in the course of the disease^23,24^. For example, presymptomatic infants treated with nusinersen in NURTURE achieved greater clinical improvement compared with symptomatic patients, as demonstrated by changes in Hammersmith Infant Neurological Examination Section 2 (HINE-2) and CHOP INTEND scores^23^.

SPR1NT enrolled infants with a genetic diagnosis of SMA, two or three copies of *SMN2*, and no clinical evidence of neuromuscular disease. The trial focused on clinically meaningful measures of efficacy, such as motor milestones compared with normal developmental benchmarks^25^ and the ability to survive and thrive without mechanical interventions, as they compared with a matched PNCR natural-history cohort^7^. Here, we report final efficacy and safety outcomes for children with two *SMN2* copies (hereafter referred to as the two-copy cohort). Fifteen children with three copies of *SMN2* (three-copy cohort) are the focus of a companion manuscript in this journal^26^. SPR1NT provides important new safety data about *SMN* gene therapy in neonates that, coupled with efficacy results from both the two-copy and three-copy cohorts, has critical implications for newborn-screening programs and the timing of therapeutic intervention.

## Results

### Screening and demographics

Forty-four newborns were screened for the SPR1NT study, and 14 in total were excluded (Supplementary Table 1). The most common reasons for exclusion were clinical signs of SMA at screening (*n* = 4), baseline peroneal nerve to tibialis anterior compound muscle action potential (CMAP) less than 2 mV (*n* = 4), and elevated anti-AAV9 titers (*n* = 2). Fourteen presymptomatic infants with genetically confirmed SMA and two *SMN2* copies (71% female) were enrolled and treated with onasemnogene abeparvovec (Supplementary Fig. 1). The first patient was enrolled on 2 April 2018, and the last patient completed the study on 4 December 2020.

Children in the two-copy cohort were born between 36 and 41 (median 38) gestational weeks, with a median weight of 3.3 kg (range, 2.72–4.35 kg) (Table 1). Eleven children were born prior to a gestational age at birth of <40 weeks (less than full-term gestation), and one patient had a gestational age of <37 weeks. All 14 children had biallelic *SMN1* deletions and two *SMN2* copies (no c.859C>G modifier variants), detected presymptomatically through either prenatal screening (*n* = 5, 36%) or newborn screening (*n* = 9, 64%). The nine children referred through newborn screening had a confirmed molecular diagnosis at a median age of 8 days (range, 1–14 days). At baseline, CHOP INTEND scores were between 28 and 57 (median 49), median peroneal CMAP was 3.9 mV (range, 2.1, 6.1 mV), and all children could swallow and breathe normally.

**Table 1:** Demographics and baseline clinical characteristics (ITT population)

Baseline characteristics	All patients (*n* = 14)
Age at dosing, days^a^	
Mean (s.d.)	20.6 (7.9)
Median (range)	21.0 (8–34)
Gestational age at birth, weeks	
Mean (s.d.)	38.2 (1.4)
Median (range)	38.0 (36–41)
Weight at baseline, kg	
Mean (s.d.)	3.6 (0.39)
Median (range)	3.7 (3.0–4.3)
Sex, *n* (%)	
Male	4 (29)
Female	10 (71)
Race, *n* (%)	
White	7 (50)
Other	4 (29)
Asian	2 (14)
Black or African American	1 (7)
American Indian or Alaska Native	0
Native Hawaiian or other Pacific Islander	0
Ethnicity, *n* (%)	
Not Hispanic or Latino	10 (71)
Hispanic or Latino	4 (29)
Modality of SMA diagnosis, *n* (%)	
Prenatal testing	5 (36)
Newborn screening	9 (64)
c.859G>C *SMN2* gene modifier variant	0
Age at SMA diagnosis, days^b^	
*n* (number of patients diagnosed after birth)	9
Mean (s.d.)	7.2 (4.8)
Median (range)	8.0 (1–14)

ITT, intention-to-treat; s.d., standard deviation; SMA, spinal muscular atrophy; *SMN2*, *survival motor neuron 2* gene.

^a^Age at dosing = (dose date – date of birth + 1).

^b^Age at SMA diagnosis = (SMA diagnosis date ‒ date of birth + 1). Only calculated for patients who were diagnosed after birth.

All 14 infants enrolled in the two-copy cohort received the entire onasemnogene abeparvovec infusion without interruption at median age 21 days of life (range, 8–34 days). All completed the study and were included in the intention-to-treat (ITT) population.

### Primary endpoint and other motor milestones

**Fig. 1:**
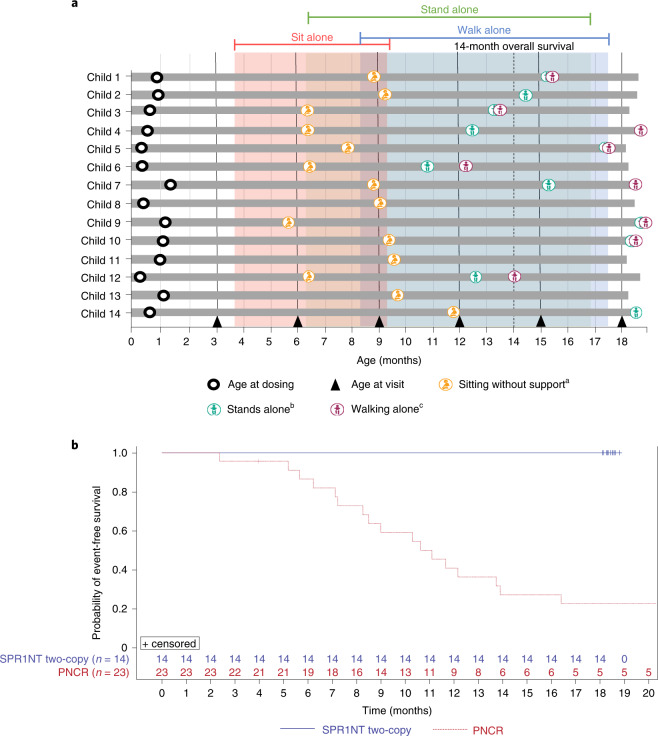
Survival and achieved video-confirmed developmental motor milestones. **a**, Milestones achieved (visit month identified). Months calculated as days/30. Only the first observed instance of a milestone is included in this figure. ^a^Bayley Scales gross motor subtest item #26: child sits alone without support for at least 30 seconds. ^b^Bayley Scales gross motor subtest item #40: child stands alone. Child stands alone for at least 3 seconds after you release his or her hands. ^c^Bayley Scales gross motor subtest item #43: child walks alone. Child takes at least five steps independently, displaying coordination and balance. According to the WHO-MGRS windows for normal development, the 99th percentile (that is, upper bound of normal development) of sitting and walking without support was 279 days and 534 days, respectively. **b**, Kaplan–Meier plot for event-free survival in the SPR1NT two-copy (blue line) and PNCR (red line) cohorts. *n* = 4 males and *n* = 10 females; mean (s.d.) age at dosing, 20.6 (7.9) days.

All 14 (100%, 97.5% confidence interval (CI): 77–100%) children in the ITT population achieved the primary endpoint of independent sitting for at least 30 seconds at any visit up to 18 months of age (Fig. 1a), compared with none of 23 untreated patients with SMA type 1 in the PNCR cohort (*P* < 0.0001). Children in the two-copy cohort first sat independently at a median age of 265 days (range, 172–354 days), and 11 of 14 (79%) achieved this motor milestone within the World Health Organization (WHO) normal developmental time window of ≤279 days of age. Of 12 children assessed for independent sitting at the end of study, all 12 (100%) retained this motor milestone at 18 months of age. The remaining two patients could not be assessed.

All 14 (100%) children achieved motor milestones as defined by both the Bayley-III Scales of Infant and Toddler Development (BSID) and the WHO Multicentre Growth Reference Study (WHO-MGRS) that were video-confirmed by an independent observer (Supplementary Tables 2 and 3). According to the BSID definition, 11 of 14 (79%) children stood alone (seven (50%) within the normal developmental window of ≤514 days).

The WHO-MGRS and BSID use slightly different criteria to describe independent walking. The WHO-MGRS defines independent walking as the ability to take five or more steps in an upright position, back straight, with one leg moving forward while the other supports most of the body weight, without contacting a person or object. BSID criteria define independent walking as the ability to take at least five steps independently, displaying coordination and balance. Nine of 14 (64%) children walked independently by BSID criteria at a median age of 526 days (range, 367–564 days), and 5 (36%) did so within the normal developmental window of ≤534 days. Ten of 14 children (71%) walked alone, as defined by WHO-MGRS criteria, at a median age of 493 days (range, 367–564 days), and six (43%) did so within the normal developmental window of ≤534 days. A comprehensive listing of motor milestone achievement is provided in Supplementary Table 4. The highest Bayley and WHO-MGRS motor milestones achieved are in Supplementary Table 5.

### Secondary endpoints

All 14 (100%) children in the two-copy cohort were alive and free of permanent ventilation at 14 months of age (first secondary endpoint), compared with 6 of 23 (26%) patients in the PNCR cohort (*P* < 0.0001) (Fig. 1b). Ventilator-free survival remained at 100% at the end of study. No child required mechanical respiratory support (for example, cough-assist, bilevel positive airway pressure, or invasive ventilatory support) of any kind throughout the duration of the trial.

**Fig. 2:**
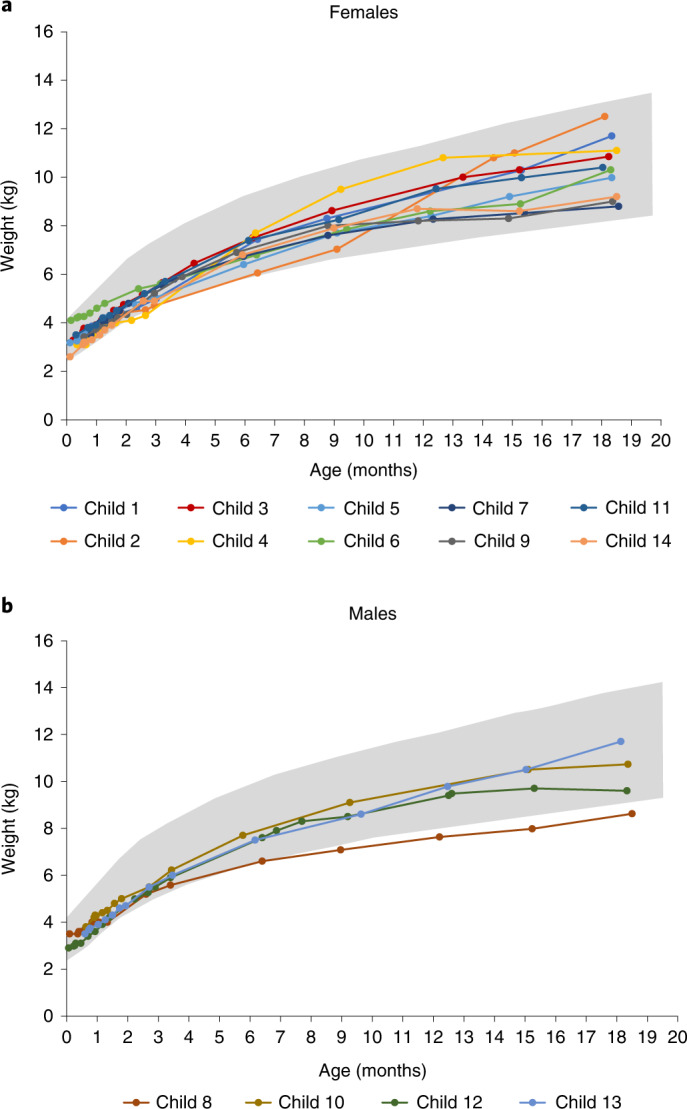
Body weight over time. Children achieved the ability to maintain weight at or greater than the 3rd percentile, without the need for non-oral or mechanical feeding support at any visit up to 18 months of age for female (**a**) and male (**b**) individuals, according to the WHO child growth standards^25^. Gray shading represents WHO growth standards for the 3rd through 97th percentiles. *n* = 4 males and *n* = 10 females; mean (s.d.) age at dosing, 20.6 (7.9) days.

Thirteen (93%) children maintained weight at or above the 3rd percentile without the need for non-oral/mechanical feeding support at all visits up to 18 months of age (second secondary endpoint, *P* < 0.0001) (Fig. 2). All 14 children (100%) remained free of non-oral or mechanical feeding support throughout the trial. Thirteen of 14 (93%) children maintained weight within an age-appropriate reference range (defined as greater than the 3rd percentile from WHO child growth standards^25^) at all study visits, and 13 of 14 (93%) tolerated thin liquids, as demonstrated through a formal swallowing test at month 18. The remaining child in the two-copy cohort was not assessed for their ability to swallow thin liquids. Ultimately, 12 (86%) children were thriving at the 18-month study visit; they could tolerate thin liquids by mouth and maintained an age-appropriate weight without mechanical feeding support (*P* < 0.0001; Supplementary Table 6).

### Exploratory endpoints

**Fig. 3:**
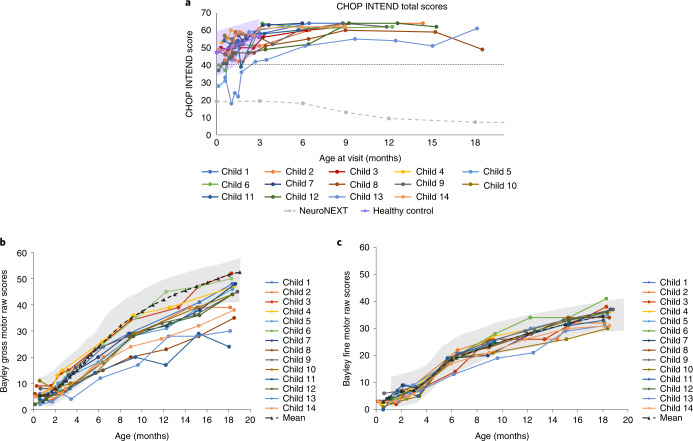
Patient-level motor function as assessed by CHOP INTEND and Bayley gross and fine motor scores. **a**, The dashed straight line represents a CHOP INTEND score of 40, which is a score that untreated patients with SMA type 1 rarely achieve in the natural history of the disease^7^. Shading represents the CHOP INTEND values obtained from normal healthy control infants in the NeuroNEXT study with the mean values presented as a solid purple line^8^. NeuroNext infants were 6 months of age or younger and born between 36–42 weeks gestation and were evaluated using the Test of Infant Motor Performance Screening Items (TIMPSI) and CHOP INTEND (for children who scored <41 on TIMPSI)^8^. The dashed gray line represents the mean change in CHOP INTEND score observed in the NeuroNEXT study of children with SMA type 1 who did not receive disease-modifying treatments^8^. Children who achieved three consecutive CHOP INTEND scores ≥58 were not tested further. Bayley scales gross motor (**b**) and fine motor (**c**) subtests. The Bayley scales gross and fine motor normal ranges (±2 s.d.) are presented in gray highlights. *n* = 4 males and *n* = 10 females; mean (s.d.) age at dosing, 20.6 (7.9) days.

In addition to documenting motor milestones, ventilator-free survival, and growth, SPR1NT included a number of other exploratory endpoints of motor function. CHOP INTEND scores (maximum score of 64) increased rapidly during the initial 3 months after onasemnogene abeparvovec infusion. Similar to the mean (s.d.) scores observed in normally developing children (47.2 (10.0) and 56.7 (5.8) at 0 and 3 months, respectively)^8^, the mean (s.d.) CHOP INTEND score for children in the two-copy cohort was 46.1 (8.8) at baseline, which increased by 3.9 (8.3) 1 month after treatment, 11.2 (8.8) at the 3 months of age visit, and 14.8 (8.1) at the visit at 6 months of age (Fig. 3a). CHOP INTEND scores reached a median of 60 (range, 51–64) by the 6-month study visit. All 14 children (100%) achieved a CHOP INTEND score greater than 40, a threshold never achieved in untreated SMA type 1 patients older than 6 months of age (*P* < 0.0001)^7,8^, whose CHOP INTEND scores instead decreased by an average 10.7 points between 6 and 12 months of age^8^. All children in the SPR1NT two-copy cohort ultimately achieved a CHOP INTEND score of at least 58 by 18 months of age (*P* < 0.0001) (Supplementary Table 7).

All 14 children (100%) demonstrated incremental gains on BSID gross and fine motor scales throughout the study duration, and all improved at least 15 points from baseline at any visit up to 18 months of age (Fig. 3b,c and Supplementary Table 8). However, BSID gross motor scores varied for children at the 18-month study visit, and five children were below the ranges (±s.d.) for normally developing children. These BSID raw scores were converted into scaled scores to allow comparison with the normative mean and distribution of BSID scores for unaffected peers. BSID-scaled scores have a normative mean of 10 and standard deviation (s.d.) of three, such that scaled scores of 4–16 represent two s.d. from the normative mean and capture the 3rd to 97th percentile range for normally developing children of similar age^27^. At one or more post-baseline visit(s), all 14 children in the two-copy cohort had a scaled score ≥4.0 (within 2 s.d. of the reference mean) on both the gross motor and fine motor BSID assessments measured at the same visit. Nine (64%) children achieved a scaled gross and fine motor score of ≥4.0 at 18 months.

Gains in motor function were paralleled by electrophysiologic evidence of improved motor nerve integrity. For 14 children in the two-copy cohort, median peroneal CMAP values increased by 0.60 mV (range, −1.3, 4.0 mV) from a median baseline of 3.9 mV (range, 2.1, 6.1), reaching a maximum post-baseline median value of 4.5 mV (range, 2.6, 6.8) (Supplementary Table 9).

### Safety endpoints

To mitigate the inflammatory response to AAV9, all 14 children commenced oral prednisolone therapy 1 day prior to onasemnogene abeparvovec infusion and completed a median of 60 (range, 49–100) days of therapy. One hundred and fifty-nine treatment-emergent AEs (TEAEs) were observed for the two-copy cohort during the study (Supplementary Tables 10 and 11). Each child experienced at least one TEAE, and five (36%) had at least one TEAE deemed to be serious. Ten of 14 (71%) had at least one TEAE considered by the investigator to be related to study treatment, but none were serious.

Five categories of AESIs were analyzed: hepatotoxicity, thrombocytopenia, cardiac toxicity, TMA, and sensory abnormalities suggestive of dorsal root ganglionopathy (Table 2). Seven hepatotoxicity AESIs occurred in three of 14 (21%) children. All events were mild or moderate, clinically asymptomatic, considered related to treatment, and resolved. One (7%) child had serum aminotransferase enzyme concentrations exceeding three times the ULN beginning on Day 352 (that is, CTCAE grade 2), and this was resolved with prednisolone dose modification (Supplementary Table 12). Two children experienced a total of four cardiac AESIs, all of which were mild or moderate elevations of creatine phosphokinase, creatine phosphokinase-MB, or troponin I that were asymptomatic and resolved with (*n* = 1) or without (*n* = 3) a temporary increase in the prednisolone dose (Supplementary Table 13). Serum cardiac troponin I was not consistently tested in all children but was elevated on four occasions for two children (maximum 0.153 μg/L on Day 13). Left ventricular ejection and shortening fractions were normal on echocardiogram, and no intracardiac thrombi were observed. Three thrombocytopenia-related AESIs occurred in three children (*n* = 1, thrombocytopenia, *n* = 1, vessel puncture site bruise, and *n* = 1, platelet count decreased), all of which were mild and resolved without intervention (Supplementary Table 14). The investigator considered two events (thrombocytopenia and platelet count decreased) as possibly related to treatment. Both events occurred on Day 8 and resolved on Days 13 and 15, respectively, with no further events reported. None of these children had platelets <75,000 cells/µL per laboratory data, and all platelet counts were within normal limits at the last assessment. No TMA events were reported during the study. Three of 14 (21%) children demonstrated areflexia (*n* = 2) and hyporeflexia (*n* = 1), both AESIs that fell within the dorsal root ganglionopathy-related criteria; however, all were mild and considered unrelated to treatment (Supplementary Table 15). Two resolved and one (areflexia) was ongoing at the end of study.

**Table 2:** Treatment-emergent adverse events of special interest (safety population)

Category of AESI preferred term	SPR1NT two-copy cohort (*n* = 14^b^) (%)
**Hepatotoxicity**	
Any TEAE	3 (21)
Aspartate aminotransferase increased	3 (21)
Alanine aminotransferase increased	1 (7)
Gamma-glutamyltransferase increased	1 (7)
**Thrombocytopenia**	
Any TEAE	3 (21)
Thrombocytopenia	1 (7)
Vessel puncture site bruise	1 (7)
Platelet count decreased	1 (7)
**Cardiac adverse events**	
Any TEAE	2 (14)
Blood creatine phosphokinase MB increased	1 (7)
Blood creatine phosphokinase increased	1 (7)
Troponin increased	1 (7)
**Sensory abnormalities suggestive of ganglionopathy**	
Any TEAE	3 (21)
Areflexia	2 (14)
Hyporeflexia	1 (7)
**Thrombotic microangiopathy**^**a**^	
Any TEAE	2 (14)
Thrombocytopenia	1 (7)
Platelet count decreased	1 (7)

AESI, adverse event of special interest; TEAE, treatment-emergent adverse event.

No TEAE representing thrombotic microangiopathy was identified.

^a^Thrombocytopenia and platelet count decreased are TEAEs that also fall under the thrombotic microangiopathy AESI category.

^b^Safety population, *n* = 4 males and *n* = 10 females; mean (s.d.) age at dosing, 20.6 (7.9) days.

## Discussion

Neonates genetically at risk for SMA type 1 who were treated in this study before 6 weeks of age, prior to symptom onset, collectively achieved developmental milestones to an extent never reported for either untreated patients with SMA type 1 or those treated with onasemnogene abeparvovec after the onset of neuromuscular symptoms. Without treatment, children with SMA type 1 never sit independently, and those with the milder SMA type 2 phenotype never achieve the ability to stand or walk. Residual motor deficits were apparent for patients treated at an older age (median, 3.5 and 4.1 months, respectively) in STR1VE-US (*n* = 22) and STR1VE-EU (*n* = 32). Only 64% of patients in STR1VE-US and 44% of patients in STR1VE-EU achieved the independent sitting endpoint, and did so at later median ages of 12.6 (US) and 15.9 (EU) months^13,14^. Only one patient from each cohort walked by age 18 months^13,14^. In contrast, children in the two-copy cohort of SPR1NT achieved remarkable gains in motor milestones: 100% sat, 71% stood, and 71% walked independently, and most did so within the normal developmental window. Exceptional motor and functional outcomes were also observed for children in the three-copy cohort of SPR1NT^26^.

For children in the two-copy SPR1NT cohort, motor gains and somatic growth nearly paralleled normal development while swallowing and respiratory function remained intact. Importantly, no child required any form of mechanical feeding or respiratory support at any time point during the trial. In comparison, 32% of symptomatic STR1VE-US patients required feeding support at some point during the study, and 18% required ventilatory support by 18 months of age^13^. Taken together, these data support the conclusion that earlier identification through systematic newborn-screening efforts and administration of onasemnogene abeparvovec prior to symptom onset results in improved developmental outcomes and greater functional independence.

Table 3 presents SPR1NT in the context of three other clinical trials, including two Phase III studies of onasemnogene abeparvovec for symptomatic infants with two copies of *SMN2* (STR1VE-US^13^ and STR1VE-EU^14^) and the Phase II study of infants with two or three copies of *SMN2* treated with nusinersen prior to symptom onset (NURTURE)^23^. Overall, Table 3 highlights the importance of treatment timing (that is, prior to the onset of clinical symptoms) as an important factor influencing outcome. However, direct comparisons are limited by differences in trial design, including primary endpoints (for example, percentage of patients who achieved ‘sits without support’ BSID item #26 milestone for the SPR1NT two-copy cohort versus time to death or respiratory intervention for NURTURE) and eligibility criteria (for example, ability to tolerate thin liquids, peroneal CMAP ≥2 mV, presymptomatic SMA type 1 or type 2 in SPR1NT versus ulnar CMAP ≥1 mV, absence of hypoxia, and no clinical signs or symptoms suggestive of SMA in NURTURE). Achievement of motor milestones in SPR1NT is also distinguished by its stringency, requiring video-confirmed assessment by an independent observer in both the two- and three-copy cohorts^26^. Regardless of these caveats, children with either few or no clinical signs of SMA who receive treatment appear to achieve more advanced developmental milestones.

**Table 3:** Summary of SPR1NT results and other SMA studies and cohorts^a^

		Onasemnogene abeparvovec				Nusinersen	
		Symptomatic patients		Presymptomatic children		Presymptomatic children	
	PNCR^7^	STR1VE-US^13^	STR1VE-EU^14^	SPR1NT two-copy cohort	SPR1NT three-copy cohort	NURTURE^b^ two-copy cohort^23^	NURTURE^b^ three-copy cohort^23^
Intention-to-treat population, *n*	23	22	32	14	15	15	10
*SMN2* copies	2	2	2	2	3	2	3
Median (range) age at diagnosis, days	N/A	67 (56–126)^c^	76 (26–156)	8 (1–14)	8 (2–26)	N/A	N/A
Median (range) age at infusion, days	N/A	105 (15–177)	123 (54–180)	21 (8–34)	32 (9–43)	19 (8–41)	23 (3–42)
Baseline median (range) CHOP INTEND	32.5 (31–33)^d^	33.5 (18–52)	28.0 (14–55)	48.5 (28–57)	N/A	45.0 (25–60)	53.5 (40–60)
Baseline median (range) CMAP amplitude, mV^e^	0.3 (0.04–1.1)	N/A	N/A	3.9 (2.1–6.1)	4.1 (2.7–7.0)	3.2 (1.1–9.7)	4.0(0.2–7.0)
Sitting independently by 18 months, *n* (%)^f^	0	14 (64)	14 (44)	14 (100)	N/A	N/A	N/A
Sitting independently by 24 months, *n* (%)^f^	0	N/A	N/A	N/A	14 (93)	15 (100)	10 (100)
Standing independently by 18 months of age, *n* (%)^f^	0	1 (5)	1 (3)	11 (79)	N/A	N/A	N/A
Standing independently by 24 months of age, *n* (%)^f^	0	N/A	N/A	N/A	15 (100)	9 (60)	10 (100)
Walking independently by 18 months, *n* (%)^f^	0	1 (5)	1 (3)	9 (64)	N/A	N/A	N/A
Walking independently by 24 months, *n* (%)^f^	0	N/A	N/A	N/A	14 (93)	9 (60)	10 (100)
Alive without permanent ventilation at 18 months, *n* (%)^f^	6 (26)^g^	20 (91)	31 (97)	14 (100)	15 (100)	15 (100)	10 (100)

N/A, not available.

^a^There are no published head-to-head studies of onasemnogene abeparvovec and nusinersen. Differences in trial design, including primary endpoints, how endpoints were measured, and eligibility criteria, make direct comparison of results from these studies infeasible. The PNCR measured CHOP INTEND, NURTURE measured WHO and HINE-2 criteria, and STR1VE-US and STR1VE-EU measured WHO criteria and CHOP INTEND.

^b^NURTURE results represent interim analysis at data cut of 29 March 2019. At the time of this analysis, the median age of the infants was 34.8 months (25.7–45.4)^23^.

^c^Median (range) is reported as the interquartile range.

^d^Values indicate median (interquartile range) obtained for patients with symptom onset <3 months of age, a group that included seven patients with two *SMN2* copies and one patient with three *SMN2* copies.

^e^Ulnar CMAP amplitude recorded from the abductor digiti minimi muscle at baseline for the PNCR study (*n* = 34 patients with SMA type 1; *n* = 23, two *SMN2* copies and *n* = 9, three *SMN2* copies) and peroneal CMAP amplitude recorded from the tibialis anterior muscle for the SPR1NT and NURTURE studies.

^f^Milestones were evaluated over different observation periods between studies, and included 18 months for STR1VE-US, STR1VE-EU, and the SPR1NT two-copy cohort, 24 months for the SPR1NT three-copy cohort, and a median follow-up time of 35 months for NURTURE.

^g^Survival without permanent ventilation at 14 months.

In contrast with the natural history of SMA type 1, motor improvements in SPR1NT were evident within 3 months of treatment, when many children had CHOP INTEND scores similar to those of healthy peers^8^. Beyond this time interval, CHOP INTEND scores for all children in the two-copy cohort remained greater than 40, a threshold never achieved by untreated patients with SMA type 1 older than 6 months of age^7,8^. All children in the two-copy cohort continued to make incremental gains on BSID gross and fine motor scales throughout the trial. These gains are also demonstrated in the three-copy cohort as reported in our companion manuscript^26^. Because motor neurons are post-mitotic, there is reason to speculate that transgene expression will be maintained in the spinal cord long-term. Accordingly, we are conducting follow-up studies to determine longitudinal motor outcomes for up to 15 years in children who participated in SPR1NT. Indeed, data from the ongoing START extension study demonstrated that newly acquired motor skills were maintained for 4.6–5.6 years after vector infusion for patients, some of whom also received nusinersen^28^. Furthermore, new motor milestones were achieved after completion of the 24-month START parent study. Neither of the patients in the therapeutic-dose cohort who achieved new milestones in the 24-month START study received nusinersen at any point^28^.

In this study, BSID gross motor scores varied for children at the 18-month study visit. These inter-individual differences in therapeutic response might, in part, reflect the extent of antenatal developmental neuropathologic changes that can result from SMN protein deficiency during fetal life^29–32^. Nevertheless, timely administration of *SMN* gene replacement prevents the rapid clinical deterioration normally observed in untreated patients with SMA type 1, likely by preventing denervation of motor units within the first 3 postnatal months^33^. In support of this idea, we observed median peroneal CMAP values of the two-copy cohort increase by 0.60 mV (range, –1.3, 4.0 mV) from baseline to the end of study. This contrasts with the age-dependent reduction in CMAP values observed in untreated patients with SMA type 1^33^.

Administration of onasemnogene abeparvovec between 8 and 34 days of age demonstrated a favorable safety profile^13,14^. TEAEs of transient elevations of liver enzymes were asymptomatic and generally mild. Platelet counts decreased transiently in a few children, but never below 75,000 cells/µL. None of the cardiac TEAEs reported were associated with clinical signs or symptoms of cardiac dysfunction, depressed cardiac function on echocardiograms, or rhythm disturbances on electrocardiograms. No cases of TMA and no events of thrombosis were reported in this study. Three children had potential TEAEs that were sensory abnormalities suggestive of dorsal root ganglionopathy: two children had areflexia and one had hyporeflexia, both of which are common features of SMA^34^. None of these children exhibited other obvious evidence of dorsal root ganglionopathy, such as painful paresthesias, sensory loss, or ataxia, although these signs may be difficult to detect in young children^35,36^. However, all potential dorsal-root-ganglionopathy-related TEAEs were considered unrelated to treatment, and two of these events resolved. The ongoing areflexia observed in one child may be reflective of underlying disease, as weak or absent deep tendon stretch reflexes are universally observed in untreated patients with SMA type 1. The favorable benefit–risk profile observed in the SPR1NT two-copy cohort is consistent with observations from patients with symptomatic SMA type 1 treated with onasemnogene abeparvovec in STR1VE-US^13^. However, no serious TEAEs related to treatment were observed in SPR1NT two-copy patients, whereas three (elevated hepatic aminotransferases in two patients and hydrocephalus in one patient) were observed in STR1VE-US^13^. Because the immune system is relatively tolerant to non-self antigens during the neonatal period^37^, it is possible that a less vigorous immune response against the vector capsid may occur in newborns.

With the availability of treatments like onasemnogene abeparvovec, there is even more urgency to identify children early in life by newborn screening and to thereby prevent death and disability by treating them presymptomatically. All children in the two-copy cohort of SPR1NT were diagnosed by either newborn screening (*n* = 9) or prenatal testing (*n* = 5) before overt signs of neuromuscular disease appeared. Presymptomatic diagnosis, when coupled with an effective therapy with acceptable risk, underscores the four Wilson and Jungner criteria^38^ most relevant to newborn screening that apply to, and are fulfilled by, SMA. These criteria are (1) an established natural history marked by significant burden of suffering and detectable preclinical phase; (2) the target population is clearly defined, including optimal timing of treatment; (3) a positive screening result triggers a consensus plan of action that includes a confirmatory testing algorithm, beneficial intervention with acceptable risk, and follow-up plan; and (4) the screening platform is robust, reproducible, and affordable at a population scale^39^. Several pilot SMA newborn-screening programs preceded SPR1NT and now comprise more than 3,700,000 neonates screened during 6 years^40–44^. These studies demonstrate that *SMN1* deletions are reliably detected from dried filter paper blood spots using high-throughput methods with excellent performance for marginal incremental cost^44–53^. They also demonstrate that some neonates, particularly those with two *SMN2* copies, develop signs of disease in the first few weeks of life^54^, consistent with several screen failures in SPR1NT. This emphasizes the urgency of timely diagnosis and treatment afforded by newborn screening. In the longer term, newborn screening coupled with presymptomatic treatment holds promise to improve health-related quality of life and reduce overall medical costs for infants otherwise expected to develop SMA type 1 ^47^. On the basis of these considerations, SMA was added to the Recommended Uniform Screening Panel in 2018 ^55,56^. As of June 2022, 46 states screen for SMA, capturing 97% of US newborns (www.curesma.org), and similar programs are taking hold worldwide.

Limitations of SPR1NT include the relatively small number of participants, the use of the PNCR external comparator group, and the exclusion of children with baseline CMAP <2 mV.

In this study, we demonstrate that onasemnogene abeparvovec, administered during the first 6 weeks post-partum to infants with biallelic *SMN1* mutations and two *SMN2* copies, but no clinical signs of SMA, alters the natural course of disease and results in better motor outcomes, ventilator-free survival, and nutritional and respiratory independence as compared with untreated patients with SMA type 1 or those treated after symptom onset. Early onasemnogene abeparvovec administration also has a favorable benefit–risk profile in presymptomatic newborns ≤6 weeks of age. To the extent these benefits endure, neonatal *SMN* gene-replacement therapy driven by systematic newborn screening efforts holds promise to ease the global burden of suffering caused by SMA type 1.

## Methods

### Study design

SPR1NT was an open-label, single-arm, Phase III study conducted at 16 sites in six countries (Australia, Belgium, Canada, Japan, the United Kingdom, and the United States). The study was conducted in accordance with the Declaration of Helsinki, International Council for Harmonisation/Good Clinical Practice guidelines, and applicable regulatory requirements (for example, those relating to informed consent and the protection of human patients in biomedical research). The study was approved by institutional review boards (IRBs) at all participating institutions (Advarra Center for IRB Intelligence, Nationwide Children’s Hospital; UCLA Medical Center IRB #3, David Geffen School of Medicine at University of California Los Angeles; Nemours Office of Human Subjects Protection, Nemours Children’s Clinic; Columbia University Medical Center IRB, Columbia University Medical Center; Advarra Center for IRB Intelligence, Massachusetts General Hospital; Children’s Hospital of Eastern Ontario Research Ethics Board, Children’s Hospital of Eastern Ontario; Sydney Children’s Hospitals Network Human Research Ethics Committee, Sydney Children’s Hospital; University of Pennsylvania IRB, Clinic for Special Children; Tokyo Women’s Medical University IRB, Tokyo Women’s Medical University Hospital; The Dubowitz Neuromuscular Centre IRB, University College London; The Neuromuscular Center of Liège, CHU & University of Liège), and written informed consent was obtained from parents or legal guardians of enrolled patients.

### Patients

The study included presymptomatic children with SMA genetically defined by biallelic deletions of *SMN1* with either two or three copies of *SMN2* who were expected to develop SMA types 1 or 2, respectively. These children were enrolled in two separate cohorts according to *SMN2* copy number. Children with *SMN1* point mutations or the *SMN2* gene modifier variant (c.859G>C) could enroll, but those with the *SMN2* gene modifier variant would not be included in the intention-to-treat (ITT) population. Efficacy and safety findings for the children with two *SMN2* copies are reported. The study planned to enroll at least 14 children with two copies of *SMN2* who met the ITT criteria and were ≤6 weeks of age at the time of gene-replacement therapy (Day 1). Full eligibility criteria are described in the Supplementary Material.

The Coronavirus Disease 2019 (COVID-19) pandemic did not impact retention. All children enrolled in SPR1NT completed the study, and none withdrew from the study or were lost to follow-up because of the COVID-19 pandemic. However, some scheduled study visits and assessments were delayed or canceled because of restrictions caused by the COVID-19 pandemic.

### Procedures

All children were admitted into the hospital for pretreatment baseline procedures 1 day prior to infusion. Onasemnogene abeparvovec (1.1 × 10^14^ vg/kg) was administered as a single intravenous infusion (given over approximately 60 minutes) between 10 April 2018 and 3 July 2019. In-patient safety monitoring was conducted for a minimum of 24 hours post-infusion. All children received prophylactic prednisolone (initially 1 mg/kg/day, increased to 2 mg/kg/day following a protocol amendment in May 2019) beginning 24 hours pre-infusion and for 48 hours post-infusion, after which the dosage was 1 mg/kg/day through a minimum of 30 days. Thereafter, prednisolone was tapered according to a standard algorithm, and based on a requirement that gamma-glutamyl transferase, alanine aminotransferase, and aspartate aminotransferase values were below the threshold of twice the ULN. Investigators were permitted to use other glucocorticosteroids in place of prednisolone, alter the daily dosage of prednisolone, and alter the taper schedule according to their clinical judgments.

Outpatient follow-up period consisted of assessments on Days 7, 14, 21, 30, 44, 51 (in Japan only), 60, and 72 post-dose, and then assessments at 3 months of age and continuing every 3 months thereafter through the 18 months of age (end of study) visit. All eligible children were invited to enroll in an ongoing long-term follow-up study (LT-002, NCT04042025).

### Outcomes

The primary efficacy endpoint was the ability to sit independently for ≥30 seconds at any visit up to 18 months of age, as stipulated by item #26 from the gross motor subtest of the BSID^27^. Secondary endpoints were survival at 14 months of age, defined as the avoidance of death or requirement of permanent ventilation (tracheostomy or ≥16 hours daily respiratory assistance for ≥14 consecutive days in the absence of an acute reversible illness, excluding perioperative ventilation) and the ability to maintain body weight at or greater than the 3rd percentile at all visits without the need for feeding support at any visit up to 18 months of age. Exploratory endpoints included achievement of motor milestones as assessed by WHO-MGRS and BSID version 3 gross motor criteria, CHOP INTEND scores, and scores on the BSID gross and fine motor subtests^27^. Videos demonstrating developmental milestones meeting WHO and BSID criteria (as part of clinical evaluation at study visits or submitted by parent(s)/legal guardian(s) at any time during the study) were reviewed by an independent, central reviewer for unbiased assessment and confirmation of developmental milestone achievement. Patients who achieved three consecutive CHOP INTEND scores ≥58 did not continue CHOP INTEND assessments. Pulmonary examinations were performed by a pulmonologist or appropriate individual as per standard institutional practice.

### Safety monitoring

Safety was assessed by monitoring for AE incidence and severity, physical examinations, pulmonary examinations, vital sign assessments, weight and length measurements, 12-lead electrocardiogram, 24-hour Holter monitoring, echocardiograms, swallowing tests, laboratory assessments, and photographs of the infusion site. TEAEs included any undesirable medical condition occurring at any time, including baseline, even if no study treatment had been administered.

All AEs were recorded and classified in accordance with the Common Terminology Criteria for Adverse Events (version 4.03) (https://www.eortc.be/services/doc/ctc/ctcae_4.03_2010-06-14_quickreference_5×7.pdf). Serious AEs occurring during the study phase met at least one of the following criteria: resulted in death; was immediately life-threatening; required an in-patient hospitalization or prolongation of existing hospitalization; resulted in a persistent or significant disability or incapacity; resulted in a congenital abnormality or birth defect; or was an important medical event that may have jeopardized the patient or required medical intervention to prevent one of the listed outcomes. The following AESIs were also analyzed: hepatotoxicity, thrombocytopenia, cardiac AEs, TMA, and sensory abnormalities suggestive of dorsal root ganglionopathy. AESIs were identified using TEAE Standardized Medical Dictionary for Regulatory Activities (MedDRA) queries and Customized MedDRA queries related to these categories (see Supplementary Methods for additional information). The relationship of AEs to onasemnogene abeparvovec (unrelated, possibly related, probably related, or definitely related) was determined by the site investigator. If there was any valid reason, even if undetermined, for suspecting a possible cause-and-effect relationship between the investigational product and the occurrence of the AE, then the AE was considered related.

### Statistical analysis

Data were analyzed using SAS version 9.4 software (SAS Institute). Primary and secondary efficacy analyses were performed for patients with biallelic *SMN1* deletions and two copies of *SMN2* without the *SMN2* gene modifier variant (c.859G>C), which is associated with a less severe clinical course^57^, who were included in the ITT population. Primary and secondary outcomes were compared with a cohort of patients from the PNCR natural-history data set (all patients with SMA type 1, two copies of *SMN2*, age at SMA onset ≤6 months, and age at SMA diagnosis ≤2 years; the *SMN2* modifier mutation (c.859G>C) was not assessed in the PNCR study cohort.)^13^. As a substitute for comparison against a rate of zero, we assumed that no more than 0.1% of untreated patients with SMA type 1 achieved independent sitting without support for ≥30 seconds up to 18 months of age or achieved the ability to maintain weight at or above the 3rd percentile without the need for non-oral/mechanical feeding support up to 18 months of age, and 26% of patients survived at 14 months according to age-matched natural-history data^7^. This study was designed to have >90% power with *α* = 0.025 to detect a significant difference in independent sitting using a one-sided exact binomial test based on a sample size of ≥14 patients into the ITT population as well as assumptions based on a matched PNCR data set^7^ and START study data^18,19^. Formal testing for the primary and secondary efficacy endpoints was performed using a hierarchical approach to protect against Type I error as follows. First, the primary endpoint of independent sitting ≥30 seconds was assessed. If the analysis of the primary endpoint was determined to be statistically significant (*P* < 0.025), then formal testing of the first secondary endpoint, percentage of patients that survived and did not require permanent ventilation, was conducted. If the analysis of this secondary endpoint was determined to be statistically significant (*P* < 0.05), then formal testing of the second secondary endpoint, maintenance of weight ≥3rd WHO percentile without feeding support at any visit up to 18 months of age, was conducted.

The safety population included all children who received onasemnogene abeparvovec, including children with *SMN1* point mutations and those with the c.859G>C *SMN2* gene modifier variant (no patients with the c.859G>C *SMN2* gene modifier variant were enrolled). Safety was evaluated through reported AEs as well as objective data variables, including vital signs, physical examinations, and laboratory studies. These data are presented in a descriptive fashion. AEs were coded using an industry standardized MedDRA coding dictionary (version 23.0), and AESIs were classified through specific predefined MedDRA terms.

### Reporting summary

Further information on research design is available in the Nature Research Reporting Summary linked to this article.

## Online content

Any methods, additional references, Nature Research reporting summaries, source data, extended data, supplementary information, acknowledgements, peer review information; details of author contributions and competing interests; and statements of data and code availability are available at 10.1038/s41591-022-01866-4.

## Supplementary information

Supplementary Information SPR1NT study group, Supplementary Methods, Supplementary Figure 1, and Supplementary Tables 1–15

Reporting Summary

## Data Availability

A redacted version of the SPR1NT study protocol and a redacted version of the statistical analysis plan are available at ClinicalTrials.gov (NCT03505099). Novartis is committed to sharing clinical trial data with external researchers and has been doing so voluntarily since 2014. Novartis is committed to sharing, upon request from qualified external researchers and subsequent approval by an independent review panel based upon scientific merit, anonymized patient-level and study-level clinical trial data, and redacted clinical study reports, for medicines and indications approved in the United States and Europe after the respective study is accepted for publication. All data provided are anonymized to respect the privacy of patients who have participated in the trial, in line with applicable laws and regulations. This trial data availability is according to the criteria and process described on www.clinicalstudydatarequest.com.
