# Supplementary material for: Onasemnogene abeparvovec for presymptomatic infants with two copies of SMN2 at risk for spinal muscular atrophy type 1: the Phase III SPR1NT trial
Source: Nat Med. 2022 Jun 17;28(7):1381–9. doi: 10.1038/s41591-022-01866-4 (PMC9205281; doi:10.1038/s41591-022-01866-4)
Supplement: Supplementary file 1 — SPR1NT study group, Supplementary Methods, Supplementary Figure 1, and Supplementary Tables 1–15 [file 41591_2022_1866_MOESM1_ESM.pdf]

nature portfolio

https://doi.org/10.1038/s41591-022-01866-4

Supplementary information

Onasemnogene abeparvovec for presymptomatic infants with two copies of *SMN2* at risk for spinal muscular atrophy type 1: the Phase III SPR1NT trial

In the format provided by the authors and unedited

Table of Contents

SUPPLEMENTARY MATERIAL **2**

SPR1NT STUDY GROUP **2**

SUPPLEMENTARY METHODS **3**

Inclusion Criteria 3

Exclusion Criteria 3

Identification of Adverse Events of Special Interest 4

SUPPLEMENTAL FIGURES **5**

Supplemental Figure 1. Patient disposition. 5

SUPPLEMENTARY TABLES **6**

Supplemental Table 1. Screen failures for the SPR1NT study 6

Supplemental Table 2. Percentages of children demonstrating Bayley Scales motor milestones up to 18 months of age (ITT population) 7

Supplemental Table 3. Percentages of children demonstrating WHO-MGRS motor milestones up to 18 months of age (ITT population) 8

Supplemental Table 4. Age at which video-confirmed developmental milestones were achieved by children during study 9

Supplemental Table 5. Highest Bayley and WHO milestones achieved (ITT population) 12

Supplemental Table 6. Percentages of children who maintain ability to thrive at 18 months of age (ITT population) 13

Supplemental Table 7. Percentages of children achieving CHOP INTEND scores up to 18 months of age (ITT population) 14

Supplemental Table 8. Summary of observed value and change from baseline in Bayley Scales for Infant and Toddler Development at each visit up to 18 months of age (ITT population) 15

Supplemental Table 9. Summary of changes from baseline to maximum post-baseline values in CMAP (ITT population) 17

Supplemental Table 10. Treatment-emergent adverse events in two or more children by preferred term and descending frequency (safety population) 18

Supplemental Table 11. Treatment-related adverse events by system organ class and preferred term (safety population) 19

Supplemental Table 12. Hepatotoxicity-related treatment-emergent adverse events of special interest (safety population) 20

Supplemental Table 13. Cardiac treatment-emergent adverse events of special interest (safety population) 21

Supplemental Table 14. Thrombocytopenia-related treatment-emergent adverse events of special interest (safety population) 22

Supplemental Table 15. Sensory abnormalities suggestive of ganglionopathy adverse events of special interest 23

SUPPLEMENTARY MATERIAL

SPR1NT STUDY GROUP

The Dubowitz Neuromuscular Centre, University College London, Great Ormond Street Institute of Child Health & Great Ormond Street Hospital, London, UK
Mariacristina Scoto

Tokyo Women's Medical University, Tokyo, Japan
Tetsuo Ikai, Tamaki Kato, Satoru Nagata, Masaki Wada, Mari Matsuo, Masaki Ogawa, Eriko Shimada, Keiko Ishigaki, Hayato Suzuki (physiotherapist), Naoko Shima (physiotherapist), Kaho Nakamura (physiotherapist)

Sydney Children's Hospital Network, Randwick, NSW, Australia
Hugo Sampaio (sub-investigator), Professor Ian E. Alexander (sub-investigator), Jonathan Forsey (cardiologist), Karen Herbert (physiotherapist), Peter Barclay (pharmacist), Nicole Kerly (study coordinator), Stephanie Richardson (study coordinator)

The Neuromuscular Center of Liège, CHU & University of Liège, Liège, Belgium
Aurore Daron and Laura Vandenbrande (co-investigators), Fabian Dal Farra (physiotherapist), Olivier Schneider (physiotherapist), Alexa Jonas (pharmacist), Laura Buscemi (study coordinator)

SUPPLEMENTARY METHODS

Inclusion Criteria

Infants eligible for enrollment in cohort 1 of SPR1NT must have been genetically diagnosed with presymptomatic spinal muscular atrophy (SMA) with two copies of *SMN2*, ≤6 weeks (≤42 days) of age at the time of treatment, were able to tolerate thin liquids as demonstrated through a formal bedside swallowing test, had a baseline tibialis anterior compound muscle action potential (CMAP) value of ≥2 mV, were at a gestational age of 35 to 42 weeks, were up-to-date on childhood vaccinations that include palivizumab prophylaxis (also known as Synagis®) to prevent respiratory syncytial virus infections, able and willing to follow the Consensus Statement for Standard of Care in Spinal Muscular Atrophy and parent(s)/legal guardian(s) willing and able to complete the informed consent process and comply with study procedures and visit schedule. Genetic diagnoses had to be obtained from an acceptable newborn or prenatal screening test method.

Exclusion Criteria

Infants were excluded from enrollment for any of the following: (1) weight at screening visit <2 kg; (2) hypoxemia (oxygen saturation <96% awake or asleep without any supplemental oxygen or respiratory support) at the screening visit or for altitudes >1000 m, oxygen saturation of <92% awake or asleep without any supplemental oxygen or respiratory support at the screening visit; (3) any clinical signs or symptoms at screening or immediately prior to dosing that were, in the opinion of the investigator, strongly suggestive of SMA (e.g., tongue fasciculation, hypotonia, areflexia); (4) tracheostomy or current prophylactic use or requirement of non-invasive ventilatory support at any time and for any duration prior to screening or during the screening period; (5) children who had signs of aspiration/inability to tolerate non-thickened liquids based on a formal swallowing test performed as part of screening or children receiving any non-oral feeding method; (6) children who had clinically significant abnormal laboratory values (gamma-glutamyl transferase, alanine aminotransferase, and aspartate aminotransferase, or total bilirubin >2× the upper limit of normal, creatinine ≥1.0 mg/dL, hemoglobin [Hgb] <8 or >18 g/dL; white blood cell [WBC] >20,000 per cmm) prior to gene replacement therapy; children with an elevated bilirubin concentration that was unequivocally the result of neonatal jaundice were not excluded; (7) children who demonstrated any other clinically significant abnormalities in hematology or clinical chemistry parameters as determined by the investigator or medical monitor; (8) children treated with an investigational or commercial product, including nusinersen, administered for the treatment of SMA; this included any history of gene therapy, prior antisense oligonucleotide treatment, or cell transplantation; (9) children whose weight-for-age was below the 3rd percentile based on World Health Organization (WHO) Child Growth Standards; (10) biologic mother with active viral infection as determined by screening laboratory samples (includes human immunodeficiency virus [HIV] or positive serology for hepatitis B or C); biological mothers with a clinical suspicion of Zika virus that met Centers for Disease Control and Prevention (CDC) Zika virus epidemiological criteria, including a history of residence in or travel to a geographic region with active Zika transmission at the time of travel were tested for Zika virus RNA; positive results warrant confirmed negative Zika virus RNA testing for the child prior to enrollment; (11) serious non-respiratory tract illness requiring systemic treatment and/or hospitalization within 2 weeks prior to screening; (12) upper or lower respiratory infection that required medical attention, medical intervention, or an increase in supportive care of any manner within 4 weeks prior to dosing; (13) severe nonpulmonary/respiratory tract infection (e.g., pyelonephritis or meningitis) within 4 weeks prior

to administration of gene replacement therapy or concomitant illness that, in the opinion of the investigator or sponsor medical monitor, created unnecessary risks for gene replacement therapy, such as major renal or hepatic impairment, known seizure disorder, diabetes mellitus, idiopathic hypocalciuria, and symptomatic cardiomyopathy; (14) known allergy or hypersensitivity to prednisolone or other glucocorticosteroids or their excipients; (15) a previous, planned, or expected major surgical procedure, including scoliosis repair surgery/procedure during the study assessment period; (16) concomitant use of any of the following: drugs for treatment of myopathy or neuropathy, agents used to treat diabetes mellitus, or ongoing immunosuppressive therapy, plasmapheresis, immunomodulators such as adalimumab, immunosuppressive therapy within 4 weeks prior to gene replacement therapy (e.g., corticosteroids, cyclosporine, tacrolimus, methotrexate, cyclophosphamide, intravenous immunoglobulin, rituximab); (17) anti-AAV9 antibody titer >1:50 as determined by enzyme-linked immunosorbent assay (ELISA) binding immunoassay; if a potential child demonstrated anti-AAV9 antibody titer >1:50, he or she received retesting inside the 30-day screening period and were eligible to participate if the anti-AAV9 antibody titer upon retesting was ≤1:50, provided child was still <6 weeks of age at the time of dosing; (18) biological mother was involved with the care of the child refused anti-AAV9 antibody testing prior to dosing; (19) parent(s)/legal guardian(s) who were unable or unwilling to comply with study procedures or the inability to travel for repeat visits; (20) parent(s)/legal guardian(s) who were unwilling to keep study results/observations confidential or to refrain from posting confidential study results/observations on social media sites; and (21) parent(s)/legal guardian(s) refused to sign consent form.

Identification of Adverse Events of Special Interest

The following specific treatment emergent adverse events of special interest (AESIs) were primarily identified by using Standardized Medical Dictionary for Regulatory Activities queries (SMQ) and Customized MedDRA queries (CMQ):

- Hepatotoxicity, identified via the following SMQ: hepatic disorders (SMQ)
- Thrombocytopenia, identified via the following CMQ: transient thrombocytopenia (CMQ)
- Cardiac events, identified via the following SMQs: ischemic heart disease (SMQ), cardiomyopathy (SMQ), cardiac arrhythmias (SMQ), embolic and thrombotic events (SMQ), and myocardial infarction (SMQ)
- Thrombotic microangiopathy, identified via the following approach:
    - Criteria #1: cases with any one of the following preferred terms (PTs): thrombotic microangiopathy OR hemolytic uremic syndrome OR atypical hemolytic uremic syndrome
    - Criteria #2: cases with at least one PT from each of the following SMQs representing thrombocytopenia, hemolysis, and relevant renal events, respectively: hematopoietic thrombocytopenia (SMQ), hemolytic disorders (SMQ), acute renal failure (SMQ) or renovascular disorders (SMQ)
- Sensory abnormalities suggestive of ganglionitis, identified via the following CMQ: DRG cell inflammation CMQ.

SUPPLEMENTAL FIGURES

Supplemental Figure 1. Patient disposition.

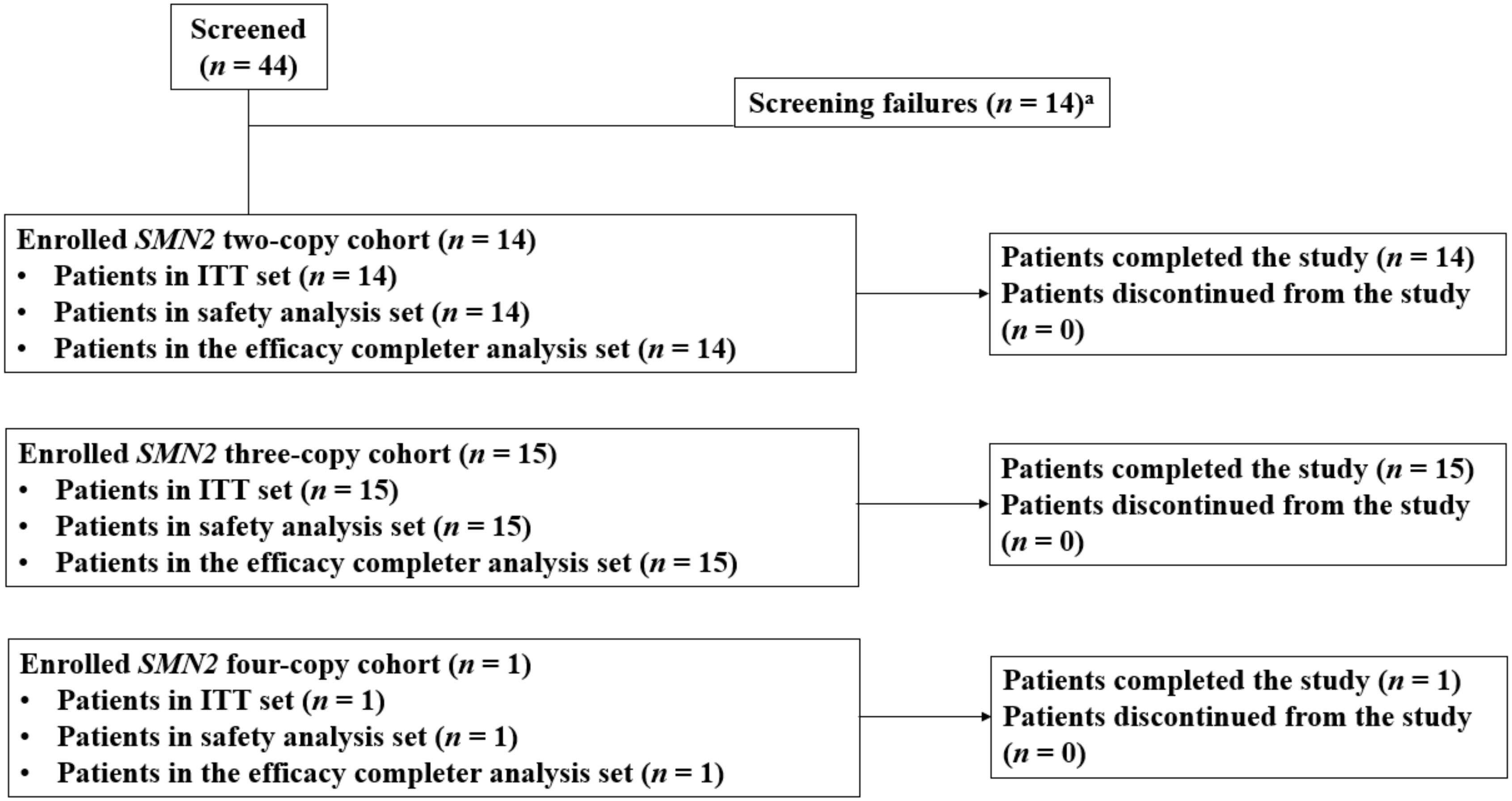

ITT, intention-to-treat; *SMN2, survival motor neuron 2* gene.
[a]Screen failures for both the two- and three-copy cohorts.

SUPPLEMENTARY TABLES

Supplemental Table 1. Screen failures for the SPR1NT study

**Criteria**	**Excluded patients (*n* = 14 total)[a]**
Clinical signs of SMA at screening	*n* = 4[b]
Baseline CMAP <2 mV	*n* = 4
Elevated anti-AAV9 titers	*n* = 2
Clinically significant abnormal laboratory parameters	*n* = 1
Signs of aspiration/inability to tolerate non-thickened liquids based on a formal swallowing test	*n* = 1
Weight for age below the 3rd percentile	*n* = 1
Lack of a genetic diagnosis, obtained from an acceptable newborn or prenatal screening test method	*n* = 1
Parent(s)/legal guardian(s) unwilling or unable to complete the informed consent process and comply with study procedures and visit schedule	*n* = 1
Serious non-respiratory tract illness requiring systemic treatment and/or hospitalization within 2 weeks prior to screening	*n* = 1

AAV9, adeno-associated virus-9; CMAP, compound muscle action potential; SMA, spinal muscular atrophy.
[a]Screen failures for both SPR1NT cohorts.
[b]Two patients had two exclusionary criteria each (clinical signs of SMA at screening and abnormal swallow for one patient and clinical signs of SMA at screening and tibialis anterior CMAP <2 mV for the other patient).

Supplemental Table 2. Percentages of children demonstrating Bayley Scales motor milestones up to 18 months of age (ITT population)

	**Two-copy cohort (*n* = 14[a])**								
	**Head control (item #4)[b]**	**Rolls from back to sides (item #20)[b]**	**Sits without support (item #26)**	**Stand with assistance (item #33)**	**Crawls (item #34)**	**Pull to stand (item #35)**	**Walks with assistance (item #37)**	**Stands alone (item #40)**	**Walks alone (item #43)**
Milestone, n/Nobs (%)	9/9 (100)	13/13 (100)	14/14 (100)	14/14 (100)	9/14 (64)	11/14 (79)	11/14 (79)	11/14 (79)	9/14 (64)
Age (days) at earliest achievement, median (95% CI)	56 (48, 86)	268 (214, 355)	265 (216, 277)	411 (314, 470)	431 (316, 446)	446 (365, 501)	376 (356, 457)	459 (402, 513)	526 (429, 549)
min, max	(35, 102)	(118, 551)	(172, 354)	(190, 564)	(268, 459)	(268, 558)	(268, 555)	(327, 564)	(367, 564)
Timing of milestone achievement[c]									
Achieved within normal range, n (%)	-	-	11 (79)	6 (43)	4 (29)	-	6 (43)	7 (50)	5 (36)
Achieved but not within normal range, n (%)	-	-	3 (21)	8 (57)	5 (36)	-	5 (36)	4 (29)	4 (29)
Did not achieve, n (%)	-	-	0	0	5 (36)	-	3 (21)	3 (21)	5 (36)

BSID, Bayley-III Scales of Infant and Toddler Development; CI, confidence interval; ITT, intention-to-treat; Nobs, number of observations; WHO-MGRS, World Health Organization Multicentre Growth Reference Study.

Percentages for each milestone achieved are based on the number of patients who did not demonstrate the milestone per the BSID assessment conducted prior to dosing (this number is represented as Nobs, or number of observations, in the table).

[a]ITT population, *n* = 4 males and *n* = 10 females; mean (SD) age at dosing, 20.6 (7.9) days.

[b]Five patients achieved head control and one achieved rolls from back to sides prior to dosing.

[c]According to the WHO-MGRS windows for normal development, the 99th percentile (i.e., upper bound of normal development window) of each milestone is as follows: sitting without support – 279 days, hands and knees crawling – 409 days, standing with assistance – 348 days, walking with assistance – 418 days, standing alone – 514 days, walking alone – 534 days.

Supplemental Table 3. Percentages of children demonstrating WHO-MGRS motor milestones up to 18 months of age (ITT population)

	Sitting without support	Hands and knees crawling	Standing with assistance	Walking with assistance	Standing alone	Walking alone
**Milestone**						
n (%)[a]	14 (100)	10 (71)	14 (100)	12 (86)	10 (71)	10 (71)
97.5% CI[b]	(77, 100)	(42, 92)	(77, 100)	(57, 98)	(42, 92)	(42, 92)
P-value[b]	<0.0001	<0.0001	<0.0001	<0.0001	<0.0001	<0.0001
**Age (days) at earliest achievement**						
Median (95% CI)	271 (230, 334)	402 (317, 448)	388 (332, 459)	447 (400, 493)	493 (439, 541)	493 (433, 537)
min, max	190, 555	250, 501	266, 555	367, 555	367, 564	367, 564
**Timing of milestone achievement[c]**						
Achieved within 99th percentile of normal development, n (%)	10 (71)	5 (36)	5 (36)	5 (36)	5 (36)	6 (43)
Achieved but not within 99th percentile of normal development, n (%)	4 (29)	5 (36)	9 (64)	7 (50)	5 (36)	4 (29)
Did not achieve, n (%)	0	4 (29)	0	2 (14)	4 (29)	4 (29)

CI, confidence interval; ITT, intention-to-treat; WHO-MGRS, World Health Organization Multicentre Growth Reference Study.
Note: Motor milestones assessed by WHO-MGRS criteria at any visit up to and including the 18 months of age visit (age ≤569 days).
[a]ITT population, *n* = 4 males and *n* = 10 females; mean (SD) age at dosing, 20.6 (7.9) days.
[b]97.5% binomial CI calculated using one-sided exact binomial test comparing observed proportion to zero. To make computation of the *P*-value possible, the value of 0.1% was used in the place of a literal 0.
[c]According to the WHO-MGRS windows for normal development, the 99th percentile (i.e., upper bound of normal development window) of each milestone is as follows: sitting without support – 279 days, hands and knees crawling – 409 days, standing with assistance – 348 days, walking with assistance – 418 days, standing alone – 514 days, walking alone – 534 days.

Supplemental Table 4. Age at which video-confirmed developmental milestones were achieved by children during study

	Age at dosing/ Age at Study completion (days)	Head control[a] Age (days/ months)	Rolls from back to sides[b] Age (days/ months)	Sits without support Age (days/months)		Crawls Age (days/months)		Stands with assistance Age (days/months)		Pulls to stand[i] Age (days/ months)	Stands alone[j,k] Age (days/ months)	Walks with assistance[l,m] Age (days/ months)	Walks alone[n,o] Age (days/ months)
				At least 30 secs[c]	At least 10 secs[d]	At least 5 feet[e]	Hands and knee crawling[f]	Supports weight 2 secs[g]	Holding stable object[h]				
Patient 1	25/550	√<br>56/1.9	√<br>193/6.4	√<br>263/8.8	√<br>263/8.8	√<br>459/15.3	√<br>459/15.3	√<br>459/15.3	√<br>459/15.3	√<br>459/15.3	√<br>459/15.3[j]<br>459/15.3[k]	√<br>459/15.3[l]<br>459/15.3[m]	√<br>459/15.3[n]<br>459/15.3[o]
Patient 2	26/559	√<br>90/3.0	√<br>194/6.5	√<br>277/9.2	√<br>277/9.2	√<br>431/14.4	√<br>431/14.4	√<br>277/9.2	√<br>375/12.5	√<br>405/13.5	√<br>431/14.4[j]<br>460/15.3[k]	√<br>375/12.5[l]<br>375/12.5[m]	√<br>544/18.1[o]
Patient 3	17/548	√<br>At screening[p]	√<br>268/8.9	√<br>188/6.3	√<br>268/8.9	√<br>268/8.9	√<br>268/8.9	√<br>268/8.9	√<br>401/13.4	√<br>268/8.9	√<br>401/13.4[j]<br>401/13.4[k]	√<br>268/8.9[l]<br>401/13.4[m]	√<br>401/13.4[n]<br>401/13.4[o]
Patient 4	19/555	√<br>50/1.7	√<br>At screening[p]	√<br>190/6.3	√<br>190/6.3	√<br>277/9.2	√<br>277/9.2	√<br>190/6.3	√<br>277/9.2	√<br>376/12.5	√<br>376/12.5[j]<br>555/18.5[k]	√<br>376/12.5[l]<br>376/12.5[m]	√<br>555/18.5[n]<br>555/18.5[o]
Patient 5	12/550	√<br>At screening[p]	√<br>273/9.1	√<br>236/7.9	273/9.1	√<br>447/14.9	√<br>372/12.4	√<br>368/12.3	√<br>447/14.9	√<br>404/13.5	√<br>526/17.5[j]<br>526/17.5[k]	√<br>447/14.9[l]<br>447/14.9[m]	√<br>526/17.5[n]<br>526/17.5[o]
Patient 6	12/549	√<br>73/2.4	√<br>118/3.9	√<br>192/6.4	√<br>192/6.4	√<br>283/9.4	√<br>250/8.3	√<br>283/9.4	√<br>283/9.4	√<br>283/9.4	√<br>327/10.9[j]<br>367/12.2[k]	√<br>367/12.2[l]<br>367/12.2[m]	√<br>367/12.2[n]<br>367/12.2[o]
Patient 7	34/559	√<br>97/3.2	√<br>266/8.9	√<br>266/8.9	√<br>266/8.9	--	--	√<br>266/8.9	√<br>266/8.9	√<br>558/18.6	√<br>460/15.3[j]<br>558/18.6[k]	√<br>369/12.3[l]<br>369/12.3[m]	√<br>558/18.6[n]<br>460/15.3[o]
Patient 8	12/556	√<br>102/3.4	√<br>192/6.4	√<br>269/9.0	√<br>555/18.5	--	--	√<br>555/18.5	√<br>555/18.5	√<br>555/18.5	--	√<br>555/18.5[l]<br>555/18.5[m]	--
Patient 9	31/564	√<br>At screening[p]	√<br>264/8.8	√<br>172/5.7	√<br>264/8.8	√<br>355/11.8	√<br>355/11.8	√<br>564/18.8	√<br>355/11.8	√<br>446/14.9	√<br>564/18.8[j]<br>564/18.8[k]	√<br>446/14.9[l]<br>446/14.9[m]	√<br>564/18.8[n]<br>564/18.8[o]
Patient 10	28/553	√<br>At screening[p]	√<br>551/18.4	√<br>278/9.3	√<br>278/9.3	√<br>453/15.1	√<br>453/15.1	√<br>453/15.1	√<br>278/9.3	√<br>453/15.1	√<br>551/18.4[j]	√<br>453/15.1[l]	√<br>551/18.4[n]

	Age at dosing/ Age at Study completion (days)	Head control[a] Age (days/ months)	Rolls from back to sides[b] Age (days/ months)	Sits without support Age (days/months)		Crawls Age (days/months)		Stands with assistance Age (days/months)		Pulls to stand[i] Age (days/ months)	Stands alone[j,k] Age (days/ months)	Walks with assistance[l,m] Age (days/ months)	Walks alone[n,o] Age (days/ months)
				At least 30 secs[c]	At least 10 secs[d]	At least 5 feet[e]	Hands and knee crawling[f]	Supports weight 2 secs[g]	Holding stable object[h]				
											551/18.4[k]	453/15.1[m]	551/18.4[o]
Patient 11	23/543	√ At screening[p]	√ 457/15.2	√ 282/9.4	√ 282/9.4	--	--	√ 457/15.2	√ 457/15.2	--	--	--	--
Patient 12	8/558	√ 35/1.2	√ 276/9.2	√ 191/6.4	√ 192/6.4	--	501/16.7	√ 244/8.1	283/9.4	--	√ 378/12.6[j] 459/15.3[k]	√ 354/11.8[l] 550/18.3[m]	√ 420/14.0[n] 420/14.0[o]
Patient 13	23/544	√ 52/1.7	√ 374/12.5	√ 289/9.6	√ 289/9.6	--	--	√ 544/18.1	√ 544/18.1	--	--	--	--
Patient 14	18/555	√ 46/1.5	√ 269/9.0	√ 354/11.8	√ 354/11.8	√ 457/15.2	√ 457/15.2	√ 555/18.5	√ 555/18.5	√ 555/18.5	√ 555/18.5[j]	√ 555/18.5[m]	--

√ = Milestone achieved (Visit month identified); developmental milestones were video confirmed by an independent central reviewer.

Note: Months calculated as days/30. Only the first observed instance of a milestone is included in this table. Per WHO-MGRS definition, months calculated as days/30.4375.

Note: *n* = 4 males and *n* = 10 females; mean (SD) age at dosing, 20.6 (7.9) days.

[a]Bayley Scales gross motor subtest item #4: Child holds head erect for at least 3 seconds without support.

[b]Bayley Scales gross motor subtest item #20: Child turns from back to both right and left sides.

[c]Bayley Scales gross motor subtest item #26: Child sits alone without support for at least 30 seconds.

[d]WHO-MGRS definition: Sitting without support. Child sits up straight with head erect for at least 10 seconds. Child does not use arms or hands to balance body or support position.

[e]Bayley Scales gross motor subtest item #34: Child makes forward progress of at least 5 feet by crawling on hands and knees.

[f]WHO MGRS definition: Hands-and-knees crawling. Child alternately moves forward or backward on hands and knees. The stomach does not touch the supporting surface. There are continuous and consecutive movements, at least three in a row.

[g]Bayley Scales gross motor subtest item #33: Supports weight. Child supports his or her own weight for at least 2 seconds, using your hands for balance only.

[h]WHO-MGRS definition: Standing with assistance. Child stands in upright position on both feet, holding onto a stable object (e.g., furniture) with both hands without leaning on it. The body does not touch the stable object, and the legs support most of the body weight. Child thus stands with assistance for at least 10 seconds.

[i]Bayley Scales gross motor subtest item #35: Child raises self to standing position using chair or other convenient object for support.

[j]Bayley Scales gross motor subtest item #40: Stands alone. Child stands alone for at least 3 seconds after you release his or her hands.

[k]WHO-MGRS definition: Standing alone. Child stands in upright position on both feet (not on the toes) with the back straight. The legs support 100% of the child's weight. There is no contact with a person or object. Child stands alone for at least 10 seconds.

[l]Bayley Scales gross motor subtest item #37: Walks with assistance. Child walks by making coordinated, alternated stepping movements.

[m]WHO-MGRS definition: Walking with assistance. Child is in upright position with the back straight. Child makes sideways or forward steps by holding onto a stable object (e.g., furniture) with one or both hands. One leg moves forward while the other supports part of the body weight. Child takes at least five steps in this manner.

[n]Bayley Scales gross motor subtest item #43: Walks alone. Child takes at least five steps independently, displaying coordination and balance.

[o]WHO-MGRS definition: Walking alone. Child takes at least five steps independently in upright position with the back straight. One leg moves forward while the other supports most of the body weight. There is no contact with a person or object.

[p]"At screening" milestone presented at baseline was not video confirmed.

Supplemental Table 5. Highest Bayley and WHO milestones achieved (ITT population)

	**Two-copy cohort (*n* = 14[a]) *n* (%)**
**Bayley Milestone**	
Stands with assistance – BSID gross motor item #33	2 (14)
Walks with assistance – BSID gross motor item #37	1 (7)
Stands alone – BSID gross motor item #40	2 (14)
Walks alone – BSID gross motor item #43	9 (64)
**WHO Milestone**	
Standing with assistance	2 (14)
Walking with assistance	2 (14)
Walking alone	10 (71)

BSID, Bayley-III Scales of Infant and Toddler Development; ITT, intention-to-treat; WHO, World Health Organization.
Motor milestones assessed by BSID or WHO criteria at any visit up to and including the 18 months of age visit (age ≤569 days).
Percentages were based on the total number of patients in the cohort. Patients were counted once according to their highest milestone.
[a]ITT population, *n* = 4 males and *n* = 10 females; mean (SD) age at dosing, 20.6 (7.9) days.

Supplemental Table 6. Percentages of children who maintain ability to thrive at 18 months of age (ITT population)

	**Statistics**	**SPR1NT two-copy (*n* = 14[a])**
Maintain ability to thrive at 18 months of age	n (%)	12 (86)
	97.5% CI[b]	(57, 98)
	P-value	<0.0001
Ability to thrive at 18 months of age[c]	*n* (%)	12 (86)
Ability to tolerate thin liquids	*n* (%)	13 (93)
Does not receive nutrition through mechanical support	*n* (%)	14 (100)
Maintains weight consistent with age	*n* (%)	13 (93)

CI, confidence interval; ITT, intention-to-treat; WHO, World Health Organization.

[a]ITT population, *n* = 4 males and *n* = 10 females; mean (SD) age at dosing, 20.6 (7.9) days.

[b]*P*-value and 97.5% CI were from a one-sided exact binomial test.

[c]The ability to thrive at month 18 was defined as meeting all of the following criteria between relative Day 540–569 : (1) the ability to tolerate thin liquids as demonstrated through a formal swallowing test, defined as follows: consistency tested was “very thin” or “thin” and the result was “normal swallow,” “functional swallow,” or “safe for swallowing”; (2) did not receive nutrition through mechanical support (i.e., feeding tube); (3) maintained weight (≥3rd percentile for age and sex as defined by WHO guidelines) consistent with the child’s age at the assessment.

Supplemental Table 7. Percentages of children achieving CHOP INTEND scores up to 18 months of age (ITT population)

**CHOP INTEND Statistics**	**SPR1NT two-copy (*n* = 14[a])**
Achieved score ≥40	
n (%)	14 (100)
97.5% CI[b]	(77, 100)
P-value	<0.0001
Achieved score ≥50	
n (%)	14 (100)
97.5% CI[b]	(77, 100)
P-value	<0.0001
Achieved score ≥58	
n (%)	14 (100)
97.5% CI[b]	(77, 100)
P-value	<0.0001

CI, confidence interval; CHOP INTEND, Children's Hospital of Philadelphia Infant Test of Neuromuscular Disorder scale; ITT, intention-to-treat.

[a]ITT population, *n* = 4 males and *n* = 10 females; mean (SD) age at dosing, 20.6 (7.9) days.

[b]97.5% binomial CI calculated using one-sided exact binomial test comparing observed proportion to zero. To make computation of the *P*-value possible, the value of 0.1% was used in the place of a literal 0.

Supplemental Table 8. Summary of observed value and change from baseline in Bayley Scales for Infant and Toddler Development at each visit up to 18 months of age (ITT population)

Visit<br>Total score		*n*	SPR1NT two-copy (*n* = 14[a]) Raw score Mean (SD)	Raw score Median (min, max)	Scaled score Mean (SD)	Scaled score Median (min, max)
**Fine motor**						
Baseline observed value		14	2.9 (1.35)	3.0 (0, 6)	8.8 (1.53)	9.0 (5, 12)
Day 30/ Month 1	Observed value	13	4.4 (2.06)	4.0 (2, 9)	9.8 (2.19)	9.0 (7, 14)
	Change from baseline	13	1.5 (2.18)	1.0 (–1, 7)	1.1 (2.5)	1.0 (–2, 8)
Month 2	Observed value	3	6.7 (2.52)	7.0 (4, 9)	11.7 (2.52)	12.0 (9, 14)
	Change from baseline	3	4.0 (2.65)	5.0 (1, 6)	3.0 (2.65)	4.0 (0, 5)
Age 3 Months	Observed value	13	7.1 (1.32)	7.0 (5, 9)	10.0 (1.68)	10.0 (7, 13)
	Change from baseline	13	4.5 (0.97)	4.0 (3, 6)	1.5 (1.20)	1.0 (0, 4)
Age 6 Months	Observed value	14	18.6 (2.47)	19.0 (13, 22)	9.1 (2.71)	10.0 (4, 13)
	Change from baseline	14	15.7 (2.87)	16.5 (10, 19)	0.4 (2.79)	1.0 (–5, 4)
Age 9 Months	Observed value	14	23.8 (2.72)	24.5 (19, 28)	8.9 (2.73)	9.5 (5, 14)
	Change from baseline	14	20.9 (3.08)	20.5 (16, 25)	0.1 (3.18)	–0.5 (–5, 5)
Age 12 Months	Observed value	10	28.1 (3.38)	28.5 (21, 34)	9.4 (3.24)	9.0 (3, 16)
	Change from baseline	10	25.1 (3.48)	25.5 (18, 31)	0.5 (3.50)	0.0 (–6, 7)
Age 15 Months	Observed value	13	31.5 (2.22)	32.0 (26, 34)	10.5 (2.15)	11 (5, 13)
	Change from baseline	13	28.6 (1.98)	29.0 (25, 31)	1.6 (2.02)	2.0 (–2, 5)
Age 18 Months	Observed value	14	34.7 (3.10)	35.0 (30, 41)	10.8 (3.26)	11.0 (6, 18)
	Change from baseline	14	31.9 (2.82)	32.0 (28, 38)	2.0 (3.04)	2.0 (–2, 9)
**Gross motor**						
Baseline	Observed value	14	5.1 (2.76)	5.0 (2, 11)	9.6 (2.21)	10 (7, 14)
Day 30/ Month 1	Observed value	14	6.0 (2.04)	6.0 (3, 9)	9.4 (1.70)	9.0 (7, 12)
	Change from baseline	14	0.9 (1.94)	1.0 (–3, 4)	–0.2 (1.63)	–0.5 (–3, 2)
Month 2	Observed value	3	10.0 (3.00)	10.0 (7, 13)	11.0 (2.65)	12.0 (8, 13)
	Change from baseline	3	6.0 (1.73)	5.0 (5, 8)	2.0 (1.00)	2.0 (1, 3)
Age 3 Months	Observed value	13	10.5 (3.04)	10.0 (6, 15)	9.2 (2.86)	8.0 (6, 14)

Visit<br>Total score		*n*	SPR1NT two-copy (*n* = 14[a])<br>Raw score<br>Mean (SD)	 <br>Median (min, max)	 Scaled score<br>Mean (SD)	 <br>Median (min, max)
	Change from baseline	13	5.7 (3.52)	5.0 (–1, 12)	–0.2 (3.17)	0.0 (–6, 5)
Age 6 Months	Observed value	14	19.4 (4.67)	19.0 (12, 26)	6.4 (3.61)	6.0 (1, 11)
	Change from baseline	14	14.3 (5.22)	15.5 (3, 23)	–3.3 (4.05)	–2.5 (-12, 4)
Age 9 Months	Observed value	14	27.5 (5.64)	28.0 (17, 36)	5.3 (3.17)	5.0 (1, 11)
	Change from baseline	14	22.4 (5.75)	23.0 (14, 33)	–4.4 (3.52)	–4.5 (-9, 3)
Age 12 Months	Observed value	10	30.8 (7.84)	31.5 (17, 45)	4.0 (3.53)	3.5 (1, 12)
	Change from baseline	10	26.6 (8.50)	26.0 (13, 43)	–5.0 (4.32)	-6.0 (–9, 5)
Age 15 Months	Observed value	13	36.8 (6.31)	38.0 (28, 47)	4.2 (3.03)	4.0 (1, 10)
	Change from baseline	13	31.7 (6.36)	33.0 (22, 45)	–5.4 (3.43)	–6.0 (–9, 3)
Age 18 Months	Observed value	14	42.1 (8.02)	44.5 (24, 52)	5.6 (3.03)	6.0 (1, 10)
	Change from baseline	14	37.1 (7.74)	39.0 (20, 48)	–4.1 (3.25)	–4.0 (–8, 2)

GM, gross motor; ITT, intention-to-treat; SD, standard deviation.
Baseline is defined as the last available assessment prior to dosing. The timing of the Month 2 visit was based on days since dosing (target: Day 60; window: Day 46 to 74) while the timing of the Month 3 visit was based on days since birth (target: age 90 days; window: age 75 to 105 days). For children whose Month 2 and Month 3 assessments fell within the Month 3 visit window, the assessment closer to the target day was used. After database lock, it was noted that the Bayley fine motor – raw and gross motor – raw scores at Month 12 for Patient 11 were not tabulated correctly by the site and are therefore not considered valid. Similarly, the Bayley GM – raw score for Patient 12, Age 18 Months Visit was not tabulated correctly by the site and therefore is not considered valid.
[a]ITT population, *n* = 4 males and *n* = 10 females; mean (SD) age at dosing, 20.6 (7.9) days.

Supplemental Table 9. Summary of changes from baseline to maximum post-baseline values in CMAP (ITT population)

	SPR1NT two-copy (n = 14[a])				
**CMAP Parameter**<br>**Visit**	***n***	**Mean (SD)**	**Median (min, max)**	**LS Mean (SE)**	***P*-value**
**CMAP amplitude (mV)[b]**					
Baseline	14	3.71 (1.228)	3.85 (2.1, 6.1)	–	
Maximum post-baseline value	14	4.66 (1.389)	4.50 (2.6, 6.8)	–	
Change from baseline	14	0.96 (1.437)	0.60 (–1.3, 4.0)	0.96 (0.354)	0.0192
**Mixed model with repeated measurements analysis[c]**					
Month 6 – observed	14	3.27 (1.212)	2.95 (1.6, 6.1)		
Month 6 – change from baseline	14	–0.44 (1.741)	–0.45 (–3.0, 4.0)	–0.47 (0.343)	0.1849
Month 12 – observed	10	3.54 (1.159)	3.50 (2.1, 5.2)		
Month 12 – change from baseline	10	–0.31 (1.521)	–0.30 (–2.2, 3.1)	–0.01 (0.359)	0.9886
Month 18 – observed	14	4.38 (1.409)	4.40 (2.5, 6.8)		
Month 18 – change from baseline	14	0.67 (1.278)	0.50 (–1.4, 2.8)	0.66 (0.343)	0.0691

CMAP, compound muscle action potential; ITT, intention-to-treat; LS, least squares; SE, standard error.

[a]ITT population, n = 4 males and n = 10 females; mean (SD) age at dosing, 20.6 (7.9) days.

[b]An analysis of covariance model was used for the analysis with maximum change from baseline in CMAP as the dependent variable and baseline value as a covariate. The maximum value for each CMAP parameter was the maximum value observed at any time post-baseline including unscheduled and out of window assessments.

[c]The statistical model is a mixed model with repeated measurements with change from baseline in CMAP as the dependent variable, fixed effect of visit, a covariate of baseline value, age at baseline (days), and interaction of baseline and visit. A Toeplitz covariance structure was used to model the within-patients errors. Only scheduled visits (Month 6, Month 12, and Month 18) are included in the model.

Supplemental Table 10. Treatment-emergent adverse events in two or more children by preferred term and descending frequency (safety population)

Preferred Term	SPR1NT two-copy (*n* = 14[a]) *n* (%)
Any TEAE	14 (100)
Pyrexia	7 (50)
Upper respiratory tract infection	5 (36)
Constipation	4 (29)
Viral upper respiratory tract infection	3 (21)
Rash	3 (21)
Hypotonia	3 (21)
Aspartate aminotransferase increased	3 (21)
Diarrhea	3 (21)
Tremor	3 (21)
Gastroesophageal reflux disease	3 (21)
Muscle contractions involuntary	3 (21)
Nasal congestion	3 (21)
Vomiting	3 (21)
Ear infection	2 (14)
Nasopharyngitis	2 (14)
Areflexia	2 (14)
Eczema	2 (14)
Influenza	2 (14)
Rhinovirus infection	2 (14)
Teething	2 (14)

TEAE, treatment-emergent adverse event.

[a]Safety population, *n* = 4 males and *n* = 10 females; mean (SD) age at dosing, 20.6 (7.9) days.

Supplemental Table 11. Treatment-related adverse events by system organ class and preferred term (safety population)

System organ class<br>Preferred term	SPR1NT two-copy<br>(*n* = 14[a])<br>*n* (%)
Children with any related TEAE[b]	10 (71)
Investigations	6 (43)
Aspartate aminotransferase increased	3 (21)
Alanine aminotransferase increased	1 (7)
Blood creatine phosphokinase MB increased	1 (7)
Blood creatine phosphokinase increased	1 (7)
Gamma-glutamyl transferase increased	1 (7)
Platelet count decreased	1 (7)
Platelet count increased	1 (7)
Troponin increased	1 (7)
Gastrointestinal disorders	5 (36)
Vomiting	3 (21)
Constipation	1 (7)
Diarrhea	1 (7)
Gastroesophageal reflux disease	1 (7)
Skin and subcutaneous tissue disorders	2 (14)
Rash	2 (14)
Blood and lymphatic system disorders	1 (7)
Thrombocytopenia	1 (7)
Eye disorders	1 (7)
Eye discharge	1 (7)
General disorders and administration site conditions	1 (7)
Malaise	1 (7)
Nervous system disorders	1 (7)
Motor developmental delay	1 (7)

TEAE, treatment-emergent adverse event.

[a]Safety population, *n* = 4 males and *n* = 10 females; mean (SD) age at dosing, 20.6 (7.9) days.

[b]TEAEs assessed by the investigator.

Supplemental Table 12. Hepatotoxicity-related treatment-emergent adverse events of special interest (safety population)

Patient ID / Age (days)[a] / Weight (kg)[b]	Preferred term	CTCAE Grade	Start/end (study day)	Outcome	Causality	Prednisolone or prednisolone equivalent dose
Patient 1/25/3.6	Aspartate aminotransferase increased	Grade 1	47/95	Recovered/ resolved	Probably related	1 mg/kg Days −1 to 34<br>0.5 mg/kg Days 35 to 48<br>0.25 mg/kg Days 49 to 84<br>Total Days=85
Patient 6/12/4.2	Aspartate aminotransferase increased	Grade 1	7/13	Recovered/ resolved	Probably related	1 mg/kg Days −1 to 30<br>0.5 mg/kg Days 31 to 44<br>0.25 mg/kg Days 45 to 58<br>Total Days=59
	Gamma-glutamyltransferase increased	Grade 1	7/13	Recovered/ resolved	Probably related	
Patient 13/23/3.7	Alanine aminotransferase increased	Grade 2	352/366	Recovered/ resolved	Possibly related	2 mg/kg Days –1 to 2<br>1 mg/kg Days 3 to 44<br>0.5 mg/kg Days 45 to 58<br>0.25 mg/kg Days 59 to 73<br>Total Days=74<br>Additional prednisolone for elevated LFTs:<br>10 mg/day Days 395 to 430<br>5 mg/day Days 431 to 433<br>2.5 mg/day Days 434 to 437
	Aspartate aminotransferase increased	Grade 2	352/366	Recovered/ resolved	Possibly related	
	Alanine aminotransferase increased	Grade 1	366/429	Recovered/ resolved	Possibly related	
	Aspartate aminotransferase increased	Grade 1	366/429	Recovered/ resolved	Possibly related	

CTCAE, Common Terminology Criteria for Adverse Events; LFT, liver function test.

Note: Safety population, n = 4 males and n = 10 females; mean (SD) age at dosing, 20.6 (7.9) days.

[a]Age at dosing (dose date − date of birth + 1).

[b]Weight at baseline.

Supplemental Table 13. Cardiac treatment-emergent adverse events of special interest (safety population)

Patient ID/age (days)[a]/weight (kg)[b]	Preferred term	CTCAE Grade	Start/end (study day)	Outcome	Causality
Patient 2/26/3.7	Troponin increased	Grade 1	7/21	Recovered/ resolved	Definitely related
	Blood creatine phosphokinase MB increased	Grade 2	13/21	Recovered/ resolved	Definitely related
	Troponin increased	Grade 1	30/42	Recovered/ resolved	Definitely related
Patient 12/8/3.0	Blood creatine phosphokinase increased	Grade 1	14/35	Recovered/ resolved	Possibly related

CTCAE, Common Terminology Criteria for Adverse Events.

Note: Safety population, *n* = 4 males and *n* = 10 females; mean (SD) age at dosing, 20.6 (7.9) days.

[a]Age at dosing (dose date – date of birth + 1).

[b]Weight at baseline.

Supplemental Table 14. Thrombocytopenia-related treatment-emergent adverse events of special interest (safety population)

**Patient ID/age (days)[a]/weight (kg)[b]**	**Preferred term**	**CTCAE Grade**	**Start/end (study day)**	**Platelets ($\times 10^9$/L)[c]**	**Outcome**	**Causality**
Patient 6/12/4.2	Vessel puncture site bruise	Grade 1	7/13	Day 7: 269<br>Normal: 160 to 370<br>Day 20: 446<br>Normal: 130 to 400	Recovered/ resolved	Unrelated
Patient 10/28/4.3	Thrombocytopenia	Grade 1	8/13	Day 8: 205<br>Day 13: 409<br>Normal: 270 to 645	Recovered/ resolved	Possibly related
Patient 11/23/3.8	Platelet count decreased	Grade 1	8/15	Day 8: 158<br>Day 15: 393<br>Normal: 270 to 645	Recovered /resolved	Possibly related

CTCAE, Common Terminology Criteria for Adverse Events.

Note: Safety population, *n* = 4 males and *n* = 10 females; mean (SD) age at dosing, 20.6 (7.9) days.

[a]Age at dosing (dose date − date of birth + 1).

[b]Weight at baseline.

[c]Platelet values for adverse event onset and resolution dates are provided. If a laboratory value was not reported on the resolution day, the value for the subsequent visit is provided. Normal values are the reference ranges used by the local or central laboratory provider, as appropriate.

Supplemental Table 15. Sensory abnormalities suggestive of ganglionopathy adverse events of special interest

**Patient ID/age (mo)[a]/weight (kg)[b]**	**Preferred term**	**CTCAE Grade**	**Start/end (study day)**	**Outcome**	**Causality**
Patient 3/17/3.6	Hyporeflexia	Grade 1	14/113	Recovered/ resolved	Unrelated
Patient 12/8/3.0	Areflexia	Grade 1	371/543	Recovered/ resolved	Unrelated
Patient 13/23/3.7	Areflexia	Grade 1	16/–	Not recovered/ not resolved	Unrelated

CTCAE, Common Terminology Criteria for Adverse Events; mo, month.

Note: Safety population, n = 4 males and n = 10 females; mean (SD) age at dosing, 20.6 (7.9) days.

[a]Age at dosing (dose date – date of birth + 1).

[b]Weight at baseline.
