# Supplementary material for: Onasemnogene abeparvovec for presymptomatic infants with two copies of SMN2 at risk for spinal muscular atrophy type 1: the Phase III SPR1NT trial
Source: Nat Med. 2022 Jun 17;28(7):1381–9. doi: 10.1038/s41591-022-01866-4 (PMC9205281; doi:10.1038/s41591-022-01866-4)
Supplement: Supplementary file 2 — Reporting Summary [file 41591_2022_1866_MOESM2_ESM.pdf]

nature research

Corresponding author(s): Kevin A. Strauss

Last updated by author(s): May 3, 2022

Reporting Summary

Nature Research wishes to improve the reproducibility of the work that we publish. This form provides structure for consistency and transparency in reporting. For further information on Nature Research policies, see our Editorial Policies and the Editorial Policy Checklist.

Statistics

For all statistical analyses, confirm that the following items are present in the figure legend, table legend, main text, or Methods section.

n/a	Confirmed	
☐	☒	The exact sample size (*n*) for each experimental group/condition, given as a discrete number and unit of measurement
☐	☒	A statement on whether measurements were taken from distinct samples or whether the same sample was measured repeatedly
☐	☒	The statistical test(s) used AND whether they are one- or two-sided<br>*Only common tests should be described solely by name; describe more complex techniques in the Methods section.*
☒	☐	A description of all covariates tested
☐	☒	A description of any assumptions or corrections, such as tests of normality and adjustment for multiple comparisons
☐	☒	A full description of the statistical parameters including central tendency (e.g. means) or other basic estimates (e.g. regression coefficient) AND variation (e.g. standard deviation) or associated estimates of uncertainty (e.g. confidence intervals)
☐	☒	For null hypothesis testing, the test statistic (e.g. *F*, *t*, *r*) with confidence intervals, effect sizes, degrees of freedom and *P* value noted<br>*Give P values as exact values whenever suitable.*
☒	☐	For Bayesian analysis, information on the choice of priors and Markov chain Monte Carlo settings
☒	☐	For hierarchical and complex designs, identification of the appropriate level for tests and full reporting of outcomes
☒	☐	Estimates of effect sizes (e.g. Cohen's *d*, Pearson's *r*), indicating how they were calculated

Our web collection on statistics for biologists contains articles on many of the points above.

Software and code

Policy information about availability of computer code

Data collection	No software was used for data collection.
Data analysis	Data were analyzed using SAS Version 9.4 software (SAS Institute).

For manuscripts utilizing custom algorithms or software that are central to the research but not yet described in published literature, software must be made available to editors and reviewers. We strongly encourage code deposition in a community repository (e.g. GitHub). See the Nature Research guidelines for submitting code & software for further information.

Data

Policy information about availability of data

All manuscripts must include a data availability statement. This statement should provide the following information, where applicable:

- Accession codes, unique identifiers, or web links for publicly available datasets
- A list of figures that have associated raw data
- A description of any restrictions on data availability

A redacted version of the SPR1NT study protocol and a redacted version of the statistical analysis plan are available at ClinicalTrials.gov (NCT03505099). Novartis is committed to sharing clinical trial data with external researchers and has been doing so voluntarily since 2014. Novartis is committed to sharing, upon requests from qualified external researchers and subsequent approval by an independent review panel based upon scientific merit, anonymized patient-level and study-level clinical trial data, and redacted clinical study reports, for medicines and indications approved in the United States and Europe after the respective study is accepted for publication. All data provided are anonymized to respect the privacy of patients who have participated in the trial in line with applicable laws and regulations. This trial data availability is according to the criteria and process described on www.clinicalstudydatarequest.com.

Field-specific reporting

Please select the one below that is the best fit for your research. If you are not sure, read the appropriate sections before making your selection.

☒ Life sciences ☐ Behavioural & social sciences ☐ Ecological, evolutionary & environmental sciences

For a reference copy of the document with all sections, see nature.com/documents/nr-reporting-summary-flat.pdf

Life sciences study design

All studies must disclose on these points even when the disclosure is negative.

Sample size	This study was designed to have >90% power with α = 0.025 to detect a significant difference in independent sitting using a one-sided exact binomial test based on a sample size of ≥14 patients into the ITT population as well as assumptions based on a matched PNCR data set and START study data. Formal testing for the primary and secondary efficacy endpoints was performed using a hierarchical approach to protect against Type I error as follows. First, the primary endpoint of independent sitting ≥30 seconds was assessed. If the analysis of the primary endpoint was determined to be statistically significant (P<0.025), then formal testing of the first secondary endpoint, percentage of patients that survived and did not require permanent ventilation, was conducted. If the analysis of this secondary endpoint was determined to be statistically significant (P<0.05), then formal testing of the second secondary endpoint, maintenance of weight ≥3rd WHO percentile without feeding support at any visit up to 18 months of age, was conducted.
Data exclusions	No data were excluded from the analyses.
Replication	This was an open-label single-arm study that included efficacy and safety assessments for each patient. Assessments were repeated for each patient at the relevant follow-up visit as per protocol, but were not replicated for each patient at each time point.
Randomization	This was an open-label single-arm study. Patients were not randomized to study groups.
Blinding	This study was an open-label design and no blinding was used.

Reporting for specific materials, systems and methods

We require information from authors about some types of materials, experimental systems and methods used in many studies. Here, indicate whether each material, system or method listed is relevant to your study. If you are not sure if a list item applies to your research, read the appropriate section before selecting a response.

Materials & experimental systems

n/a	Involved in the study	
☒	☐	Antibodies
☒	☐	Eukaryotic cell lines
☒	☐	Palaeontology and archaeology
☒	☐	Animals and other organisms
☐	☒	Human research participants
☐	☒	Clinical data
☒	☐	Dual use research of concern

Methods

n/a	Involved in the study	
☒	☐	ChIP-seq
☒	☐	Flow cytometry
☒	☐	MRI-based neuroimaging

Human research participants

Policy information about studies involving human research participants

Population characteristics	Infants eligible for enrollment in the two-copy cohort of SPR1NT must have been genetically diagnosed with presymptomatic spinal muscular atrophy (SMA) with two copies of SMN2, ≤6 weeks (≤42 days) of age at the time of treatment, were able to tolerate thin liquids as demonstrated through a formal bedside swallowing test, had a baseline peroneal nerve to tibialis anterior compound muscle action potential (CMAP) value of ≥2 mV, were at a gestational age of 35 to 42 weeks, were up-to-date on childhood vaccinations that include palivizumab prophylaxis (also known as Synagis®) to prevent respiratory syncytial virus infections, able and willing to follow the Consensus Statement for Standard of Care in Spinal Muscular Atrophy and parent(s)/legal guardian(s) willing and able to complete the informed consent process and comply with study procedures and visit schedule. Genetic diagnoses had to be obtained from an acceptable newborn or prenatal screening test method.<br><br>Fourteen presymptomatic infants with genetically confirmed SMA and two SMN2 copies (71% female) were enrolled and treated with onasemnogene abeparvovec. Children in the two-copy cohort were born between 36 and 41 (median 38) gestational weeks, with a median weight of 3.3 kg (range, 2.72–4.35 kg). Eleven children were born prior to a gestational age at birth of <40 weeks (less than full-term gestation), and one patient had a gestational age of <37 weeks. All 14 children had biallelic SMN1 deletions and two SMN2 copies (no c.859C>G modifier variants) detected presymptomatically through either

	prenatal screening (n = 5, 36%) or newborn screening (n = 9, 64%). The nine children referred by newborn screening had a confirmed molecular diagnosis at median age 8 days (range, 1–14 days).
Recruitment	Infants eligible for enrollment in the two-copy cohort of SPR1NT must have been genetically diagnosed with presymptomatic spinal muscular atrophy (SMA) with two copies of SMN2, ≤6 weeks (≤42 days) of age at the time of treatment, were able to tolerate thin liquids as demonstrated through a formal bedside swallowing test, had a baseline peroneal nerve to tibialis anterior compound muscle action potential (CMAP) value of ≥2 mV, were at a gestational age of 35 to 42 weeks, were up-to-date on childhood vaccinations that include palivizumab prophylaxis (also known as Synagis®) to prevent respiratory syncytial virus infections, able and willing to follow the Consensus Statement for Standard of Care in Spinal Muscular Atrophy and parent(s)/legal guardian(s) willing and able to complete the informed consent process and comply with study procedures and visit schedule. Genetic diagnoses had to be obtained from an acceptable newborn or prenatal screening test method.<br><br>SMA is a rare disease, and the study was conducted at specialized tertiary care centers. There were no active recruitment efforts that would create bias.
Ethics oversight	The study was approved by institutional review boards at all participating institutions (Advarra Center for IRB Intelligence, Nationwide Children's Hospital; UCLA Medical Center IRB #3, David Geffen School of Medicine at University of California Los Angeles; Nemours Office of Human Subjects Protection, Nemours Children's Clinic; Columbia University Medical Center IRB, Columbia University Medical Center; Advarra Center for IRB Intelligence, Massachusetts General Hospital; Children's Hospital of Eastern Ontario Research Ethics Board, Children's Hospital of Eastern Ontario; Sydney Children's Hospitals Network Human Research Ethics Committee, Sydney Children's Hospital; University of Pennsylvania IRB, Clinic for Special Children; Tokyo Women's Medical University IRB, Tokyo Women's Medical University Hospital; The Dubowitz Neuromuscular Centre IRB, University College London; The Neuromuscular Center of Liège, CHU & University of Liège), and written informed consent was obtained from parents or legal guardians of enrolled patients.

Note that full information on the approval of the study protocol must also be provided in the manuscript.

Clinical data

Policy information about clinical studies

All manuscripts should comply with the ICMJE guidelines for publication of clinical research and a completed CONSORT checklist must be included with all submissions.

Clinical trial registration	ClinicalTrials.gov NCT03505099, registration date April 23, 2018.
Study protocol	A redacted version of the SPR1NT study protocol and a redacted version of the statistical analysis plan are available at ClinicalTrials.gov.
Data collection	Data collection began between September 18, 2018, and July 9, 2019, at the time of onasemnogene abeparvovec infusion, and the patients were followed for 18 months. Data collection was undertaken at 16 sites in six countries (Australia, Belgium, Canada, Japan, United Kingdom, and the United States of America).
Outcomes	The primary efficacy endpoint was the ability to sit independently for ≥30 seconds at any visit up to 18 months of age, as stipulated by item #26 from the gross motor subtest of the Bayley-III Scales of Infant and Toddler Development (BSID). Secondary endpoints were survival at 14 months of age, defined as the avoidance of death or requirement of permanent ventilation (tracheostomy or ≥16 hours daily respiratory assistance for ≥14 consecutive days in the absence of an acute reversible illness, excluding perioperative ventilation) and the ability to maintain body weight at or greater than the 3rd percentile at all visits without the need for feeding support at any visit up to 18 months of age. Exploratory endpoints included achievement of motor milestones as assessed by WHO Multicentre Growth Reference Study (WHO-MGRS) and BSID Version 3 Gross Motor criteria, Children's Hospital of Philadelphia Infant Test of Neuromuscular Disorders (CHOP INTEND) scores, and scores on the BSID gross and fine motor subtests. Videos demonstrating developmental milestones meeting WHO and BSID criteria (as part of clinical evaluation at study visits or submitted by parent(s)/legal guardian(s) at any time during the study) were reviewed by an independent, central reviewer for unbiased assessment and confirmation of developmental milestone achievement. Patients who achieved three consecutive CHOP INTEND scores ≥58 did not continue CHOP INTEND assessments.<br><br>Primary and secondary efficacy analyses were performed for patients with biallelic SMN1 deletions and two copies of SMN2 without the SMN2 gene modifier variant (c.859G>C), which is associated with a less severe clinical course, who were included in the ITT population. Primary and secondary outcomes were compared with a cohort of patients from the PNCR natural history data set described (all patients with SMA type 1, two copies of SMN2, age at SMA onset ≤6 months, and age at SMA diagnosis ≤2 years; the SMN2 modifier mutation [c.859G>C] was not assessed in the PNCR study cohort.). As a substitute for comparison against a rate of zero, we assumed that no more than 0.1% of untreated patients with SMA type 1 achieved independent sitting without support for ≥30 seconds up to 18 months of age or achieved the ability to maintain weight at or above the 3rd percentile without the need for non-oral/mechanical feeding support up to 18 months of age, and 26% of patients survived at 14 months according to age-matched natural history data. This study was designed to have >90% power with $\alpha = 0.025$ to detect a significant difference in independent sitting using a one-sided exact binomial test based on a sample size of ≥14 patients into the ITT population as well as assumptions based on a matched PNCR data set and START study data. Formal testing for the primary and secondary efficacy endpoints was performed using a hierarchical approach to protect against Type I error as follows. First, the primary endpoint of independent sitting ≥30 seconds was assessed. If the analysis of the primary endpoint was determined to be statistically significant ($P<0.025$), then formal testing of the first secondary endpoint, percentage of patients that survived and did not require permanent ventilation, was conducted. If the analysis of this secondary endpoint was determined to be statistically significant ($P<0.05$), then formal testing of the second secondary endpoint, maintenance of weight ≥3rd WHO percentile without feeding support at any visit up to 18 months of age, was conducted.
